# The Interplay Between PMOS and MASLD: Pathophysiology and Evidence-Based Nutritional Interventions

**DOI:** 10.3390/nu18121948

**Published:** 2026-06-16

**Authors:** Ashley Graef, Monique J. LeMieux

**Affiliations:** Nutrition and Food Sciences Department, Texas Woman’s University, Denton, TX 76204, USA

**Keywords:** polycystic ovary syndrome, polyendocrine metabolic ovarian syndrome, metabolic dysfunction-associated steatotic liver disease, insulin resistance, dietary interventions, omega-3 fatty acids, myo-inositol, Mediterranean diet, narrative review

## Abstract

Background: Polyendocrine Metabolic Ovarian Syndrome (PMOS) is recognized as the most prevalent endocrine disorder among women of reproductive age, with an estimated prevalence of approximately 18% according to current Rotterdam diagnostic criteria. Conditions such as dyslipidemia, insulin resistance, hyperandrogenism, central adiposity, and chronic inflammation are frequently observed in women diagnosed with PMOS. These conditions predispose such individuals to an increased risk of developing Metabolic Dysfunction-Associated Steatotic Liver Disease (MASLD), which is the most prevalent chronic liver disease worldwide, affecting approximately 25% of the global population. PMOS and MASLD represent two interconnected metabolic disorders that share overlapping risk factors. Objective: The purpose of this narrative review is to investigate the underlying pathophysiological connections between PMOS and MASLD and to assess the efficacy of targeted nutritional interventions. Methods: An analysis of nearly 30 articles concerning nutritional strategies for PMOS and MASLD was conducted, including studies on dietary patterns, macronutrient-focused dietary strategies, and dietary supplement interventions. Results and Conclusions: The review concludes that a combined approach—comprising an anti-inflammatory dietary pattern, omega-3 supplementation, and myo-inositol—serves as a good evidence-based initial strategy for clinicians and dietitians managing women with coexisting PMOS and MASLD. It is noteworthy that most of the evidence for these recommendations comes from studies that focus solely on either PMOS or MASLD populations. Studies involving individuals with both conditions are currently lacking. Future research should prioritize long-term randomized controlled trials involving women from diverse backgrounds diagnosed with both PMOS and MASLD. These conditions, whether independently or concurrently, are imposing an increasing burden on women of reproductive age worldwide. As further research is conducted, nutritional interventions may serve as primary rather than supplementary therapeutic strategies for the management of PMOS and MASLD.

## 1. Introduction

Polyendocrine Metabolic Ovarian Syndrome (PMOS), formerly known as Polycystic Ovary Syndrome (PCOS), and Metabolic Dysfunction-Associated Steatotic Liver Disease (MASLD), previously referred to as Non-Alcoholic Fatty Liver Disease (NAFLD), are two interconnected metabolic disorders characterized by similar features.

PMOS is a prevalent endocrine disorder that impacts women during their reproductive years, marked by a disruption in reproductive hormone balance, resulting in a spectrum of symptoms and health challenges. PMOS is characterized by irregular menstrual cycles, hyperandrogenism, hirsutism, thinning hair on the scalp, weight gain or obesity, and polycystic ovaries [1,2]. Depending on the criteria used, it affects 5–28% of women of reproductive age [3]. The most common diagnostic criteria are the Rotterdam criteria, which require at least two symptoms from the following: hyperandrogenism, ovulatory dysfunction, or polycystic ovaries [3,4]. The etiology of PMOS is unknown; however, it is likely a combination of genetic and environmental factors [3]. PMOS is also associated with various metabolic abnormalities, including insulin resistance (IR), type 2 diabetes mellitus (T2DM), obesity, dyslipidemia, and an increased risk of heart disease [3,5].

MASLD, defined by the presence of one of five cardiometabolic risk factors in the context of hepatic steatosis, has emerged as the prevailing chronic liver ailment globally, affecting approximately a quarter of the world’s population [6,7]. MASLD refers to a spectrum of liver conditions marked by the accumulation of excess fat in liver cells among individuals who do not consume excessive amounts of alcohol. It ranges from simple steatosis (fatty liver), which is generally benign and has a lower risk of progression, to metabolic dysfunction-associated steatohepatitis (MASH). This more severe form includes liver inflammation and can progress to advanced fibrosis, cirrhosis, and even hepatocellular carcinoma in rare instances [8]. MASLD is the most common chronic liver condition in many parts of the world [8]. Diagnosis typically depends on evidence of hepatic steatosis, either by imaging or histology, a history of no or limited alcohol intake, and exclusion of other causes of liver disease [9].

Research has shown a strong association between PMOS and MASLD, with both conditions sharing common pathophysiological mechanisms, including IR, hyperandrogenemia, obesity, and dyslipidemia [5,10,11]. Furthermore, the incidence of MASLD in women with PMOS is higher (34–70%) compared to women without PMOS (14–34%). The inverse is also true, in that women who are diagnosed with MASLD are often diagnosed with PMOS as well [12]. This relationship suggests that PMOS may be a significant risk factor for the development of MASLD, particularly in women of reproductive age [11]. The presence of MASLD in women with PMOS can exacerbate hormonal disorders and increase the risk of cardiometabolic complications [11].

Despite these links, research focusing specifically on women with PMOS and MASLD remains limited. Additionally, the literature highlights a pressing need not only to deepen the understanding of PMOS-MASLD pathophysiology but also to connect this knowledge with evidence on nutritional interventions. Therefore, this narrative review aims to explore the physiological relationship between PMOS and MASLD and evaluate the effectiveness of targeted nutritional strategies. As one of the first reviews to compile evidence on nutritional treatments at the intersection of these conditions within the updated MASLD and PMOS frameworks, it emphasizes common findings and highlights remaining gaps. The findings aim to serve as a practical, evidence-based resource for dietitians and healthcare providers managing women with these disorders.

## 2. Pathophysiological Mechanisms Linking PMOS and MASLD

**Figure 1:**
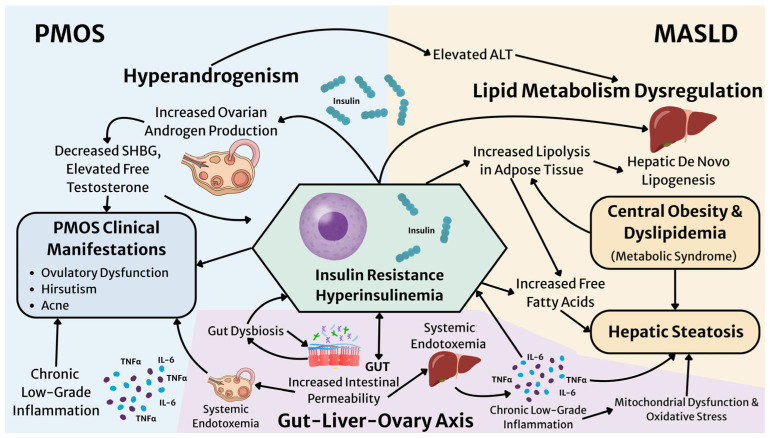
Pathophysiological links and shared risk factors between PMOS and MASLD. Shared characteristics of metabolic syndrome, notably central obesity and dyslipidemia, constitute the fundamental bidirectional link among insulin resistance, chronic low-grade inflammation, and hyperandrogenism in polyendocrine metabolic ovarian syndrome (PMOS) and metabolic dysfunction-associated steatotic liver disease (MASLD). Insulin resistance and pro-inflammatory cytokines such as IL-6 and TNF-α promote both hepatic steatosis and ovarian dysfunction. This cellular interaction is further modulated by the emerging gut–liver–ovary axis, in which gut dysbiosis and increased intestinal permeability exacerbate systemic inflammation and metabolic disturbances in both organs. Figure created using Canva Pro (Canva Pty Ltd., Sydney, Australia).

The pathophysiological link between PMOS and MASLD involves IR, hyperandrogenism, and chronic low-grade inflammation, which are all common features in both conditions (Figure 1). Byrne and Targher discuss how MASLD is a multisystem disease that affects not only the liver but also other systems, potentially worsening the metabolic disturbances seen in PMOS [9]. This includes the risk of T2DM, cardiovascular diseases (CVD), and chronic kidney disease, which are also prevalent in PMOS populations [9].

### 2.1. Insulin Resistance

Insulin resistance (IR) is a key factor in the development of both PMOS and MASLD, serving as a shared link between these conditions. IR affects about 65–70% of women with PMOS [13,14], increasing their risk for T2DM and gestational diabetes [15]. In PMOS, IR promotes hyperandrogenism, a central feature of the syndrome. This hyperandrogenism further worsens IR, forming a vicious cycle that can lead to various metabolic issues [3]. As a result, IR-driven hyperinsulinemia—both basal and glucose-stimulated—raises ovarian androgen production and reduces SHBG (sex hormone-binding globulin) synthesis, worsening hypothalamic–pituitary–ovarian and systemic dysfunctions [16,17] (Figure 1). Although obesity [18] is linked to impaired glucose regulation, this dysregulation is frequently seen in women with PMOS regardless of body mass index (BMI) [19].

IR is also a central feature in the development and progression of MASLD. It promotes hepatic lipid accumulation and increases the risk of progression from simple steatosis to more severe forms of MASLD, such as MASH [9] (Figure 1). The presence of PMOS further complicates this scenario, as the syndrome itself is associated with a higher risk of developing MASLD. Women with PMOS have a significantly higher prevalence of MASLD compared to those without PMOS, and this relationship is influenced by factors like hyperandrogenism and obesity, which are common in PMOS and contribute to IR [20]. The interplay between PMOS and MASLD, mediated by IR, suggests that managing insulin sensitivity could be crucial in treating or mitigating both conditions. Therapies enhancing insulin sensitivity, such as Metformin, have demonstrated beneficial effects in PMOS patients by reducing hyperandrogenism and improving ovulatory functions. Similarly, managing IR could potentially alleviate the hepatic burden in MASLD and reduce the progression of liver disease [3,9].

### 2.2. Hyperandrogenism

Hyperandrogenism, a hallmark of PMOS, is characterized by elevated levels of androgens such as testosterone. This condition not only contributes to the reproductive symptoms of PMOS but also plays a significant role in metabolic dysfunctions associated with the syndrome, including IR [3]. Hyperandrogenism exacerbates IR, which in turn can lead to an increased risk of developing MASLD [20]. In PMOS, hyperandrogenism is associated with MASLD development regardless of obesity. Elevated blood ALT levels in women with PMOS and hyperandrogenism indicate that excess androgens might negatively affect the liver [21]. Furthermore, women with PMOS and hyperandrogenism face a notably higher risk of MASLD than those without it, implying that androgen excess may directly cause liver damage through processes that promote lipid accumulation and inflammation [20] (Figure 1). The role of IR further complicates the interplay between hyperandrogenism and MASLD. IR associated with hyperandrogenism in PMOS can promote hepatic lipid accumulation, thus exacerbating MASLD [9]. Managing hyperandrogenism in PMOS patients, therefore, might not only help in alleviating the reproductive and metabolic symptoms of PMOS but could also potentially reduce the risk or severity of MASLD.

### 2.3. Chronic Low-Grade Inflammation

Chronic low-grade inflammation plays a pivotal role in the development and progression of both PMOS and MASLD, exacerbating IR and contributing to the clinical manifestations of these conditions (Figure 1). Aside from IR, one of the most prominent features of PMOS is chronic low-grade inflammation [22]. The presence of chronic low-grade inflammation in PMOS is evidenced by elevated levels of inflammatory markers, including C-reactive protein (CRP), tumor necrosis factor-alpha (TNFα), and interleukins (e.g., IL-6). These markers of inflammation impair insulin action and are associated with the metabolic disturbances observed in these conditions PMOS [5,23]. Similarly, MASLD is associated with an inflammatory state that contributes to the progression from simple steatosis to more severe forms of liver disease, such as MASH. In MASLD, the accumulation of fat in liver cells leads to increased oxidative stress and the release of pro-inflammatory cytokines, which in turn promote inflammation and fibrosis in the liver [5,23]. Shared inflammatory pathways may partially explain the link between PMOS and MASLD. Both conditions are associated with obesity, which itself is a pro-inflammatory state. Adipose tissue in obese individuals, including those with PMOS and MASLD, secretes various adipokines that contribute to systemic inflammation and IR [5,23].

### 2.4. Gut–Liver–Ovary Axis

Emerging research suggests a potential role for the gut microbiome in metabolic diseases. Overall, the gut–liver–ovary axis in PMOS describes a vicious cycle where gut dysbiosis increases intestinal permeability, allowing endotoxins such as lipopolysaccharide (LPS) to enter the circulation. This promotes liver inflammation, IR, and hyperandrogenism, ultimately leading to ovarian dysfunction (Figure 1). This axis links chronic inflammation, hormonal imbalances, and metabolic factors disturbances [24,25,26,27,28,29]. Patients with PMOS often exhibit decreased alpha and beta microbial diversity, along with increased levels of *Firmicutes*, which are associated with metabolic disorders such as obesity and MASLD [24,25,26]. This dysbiosis harms the intestinal barrier, allowing toxins such as LPS to induce liver inflammation and fat accumulation, thereby promoting IR [26,27,28,29]. Further research into the gut–liver and gut–ovary axes could shed light on how microbiome changes influence the development and progression of PMOS and MASLD and may help identify new therapeutic targets [6,9].

## 3. Shared Risk Factors and Screening of PMOS and MASLD

### 3.1. Metabolic Syndrome Components

Metabolic syndrome (MetS) is a complex disorder characterized by a group of interconnected factors that promote a pro-inflammatory and prothrombotic metabolic state, increasing the risk of developing T2DM and/or CVD [30,31]. The main components of MetS include dyslipidemia, hypertension, glucose intolerance, and excess abdominal obesity [30]. While metabolic syndrome is not an absolute risk indicator, about 30% to nearly 50% of women with PMOS also meet the diagnostic criteria for MetS [32,33,34,35]. This rate is three to five times higher than that of women without PMOS [32,33,34,36,37]. The most common components of MetS in women with PMOS are low high-density lipoprotein cholesterol (HDL-C, up to 84.9%), central obesity (up to 56.6%), and high triglycerides (TG, 44.33%) [33]. MASLD is strongly associated with MetS and is often considered the liver-related expression of MetS [38]. Studies indicate that between 43.2% and 53% of individuals with MetS have MASLD, compared to approximately 18–25% in the general population [39,40]. Not only does MetS lead to MASLD, but studies also indicate that MASLD may promote the development of MetS [38]. Increased energy intake relative to expenditure can lead to ectopic fat accumulation in the liver, thereby increasing hepatic gluconeogenesis and promoting the development of IR [38]. This section examines two components of MetS, namely, obesity and dyslipidemia, and their respective roles in PCOS and MASLD.

#### 3.1.1. Obesity

Obesity is a significant risk factor for both PMOS and MASLD. In PMOS, obesity worsens the metabolic and reproductive irregularities linked to the syndrome. It is linked to increased IR, which then contributes to hyperandrogenism [23]. Similarly, obesity is strongly associated with MASLD, as it promotes IR and increases the risk of developing more severe liver conditions such as MASH and cirrhosis [5,23] (Figure 1). The presence of obesity in PMOS patients significantly increases the risk of developing MASLD. Studies have shown that the prevalence of MASLD is markedly higher in individuals with obesity, reaching up to 90% in patients with morbid obesity [23]. Another study also found that the prevalence of MASLD was significantly higher in women with PMOS and obesity compared to women with PMOS alone [5,23]. Furthermore, central adiposity, or the accumulation of fat in the abdominal area, is particularly relevant in the context of MASLD and PMOS. Central adiposity is more closely associated with metabolic complications than overall obesity and is a common feature in PMOS. This type of fat distribution contributes to increased IR and is an important regulator of fatty liver in MASLD [23].

#### 3.1.2. Dyslipidemia

Dyslipidemia is a common feature in both PMOS and MASLD, often contributing to the cardiovascular risks associated with these conditions. In PMOS, dyslipidemia manifests as elevated total cholesterol (TC), low-density lipoprotein cholesterol (LDL-C), and TGs, along with reduced HDL-C. This lipid profile contributes to the increased risk of CVD observed in PMOS patients [41]. Similarly, MASLD is associated with metabolic dysregulations, including dyslipidemia. The condition is frequently accompanied by elevated TGs and LDL-C, and reduced HDL-C. These lipid abnormalities in MASLD are also linked to an increased risk of CVD [41]. Patients with MASLD also exhibit dysregulated cholesterol metabolism. Those with more advanced stages produce increased endogenous cholesterol but face difficulty in efficiently removing cholesterol from liver pools, causing accumulation [42]. Similarly, the dyslipidemia and hypercholesterolemia observed in PMOS likely result from alterations in cholesterol metabolism, as both conditions share the same pathways.

### 3.2. Genetic Influences and Epidemiological Connections

Genetic factors play a significant role in the association between MASLD and PMOS. Liu et al. [43] conducted a Mendelian randomization study that found that individuals with higher genetic liability to MASLD were more likely to develop PMOS. This study used genome-wide association study (GWAS) data to identify genetic instruments that suggest a causal relationship between MASLD and PMOS. The study also highlighted the mediating roles of glycemic-related traits and sex hormones in this association, suggesting that IR and serum androgen levels might be critical mediators in the hepato-ovarian axis linking MASLD and PMOS [43]. Chen et al. [44] also explored the genetic associations between MASLD and PMOS by identifying differentially expressed genes and constructing a protein–protein interaction network. This study highlighted several hub genes, such as TREM1, S100A9, FPR1, NCF2, FCER1G, CCR1, S100A12, MMP9, and IL1RN, which were significantly upregulated in both conditions. These genes are involved in immune response and inflammatory processes, suggesting that inflammation may be a common genetic pathway linking MASLD and PMOS. Together, these studies provide evidence of genetic links between MASLD and PMOS, highlighting the importance of genetic susceptibility, inflammation, and metabolic traits in understanding the pathophysiology of these conditions.

Numerous epidemiological studies have established a higher prevalence of MASLD in women with PMOS when compared to the general population. The findings from Asfari et al. indicate a significant association between the two disorders, with women with PMOS being four times more likely to develop MASLD compared to women without PMOS [45]. The study’s use of national databases and its large scale support the hypothesis that PMOS contributes to the risk of developing MASLD. Radenkovic et al. highlight that the presence of MASLD is increased in women with PMOS, especially those with high serum androgen levels, obesity, and IR. The review emphasizes that these conditions have multifactorial points of contact, suggesting a complex interplay rather than a full coexistence. The study also notes that IR plays a pivotal role in the pathogenesis of both PMOS and MASLD, thereby linking these two conditions [46]. Shahbaz et al. support these findings by stating that all studies included in their systematic review showed a higher incidence of MASLD among individuals with PMOS in comparison to those without the condition. The review specifically points out that hyperandrogenism is the most influential risk factor for the development of MASLD in these patients [47]. This is consistent with the observations in Radenkovic et al. [46], where hyperandrogenism, along with IR and obesity, is noted as a significant contributor to MASLD in women with PMOS.

### 3.3. Current Diagnosis and Screening Recommendations

At present, there is no universal diagnostic standard for PMOS. Over the years, significant efforts have been made to define and diagnose PMOS, but consensus remains elusive. The first formal attempt was made by the US National Institutes of Health (NIH) in April 1990, which required women to have both hyperandrogenism and chronic oligo-anovulation for diagnosis. The second effort came in May 2003 when the European Society for Human Reproduction and Embryology and the American Society for Reproductive Medicine (ESHRE/ASRM) convened in Rotterdam, the Netherlands. This set of criteria, known as the Rotterdam criteria, broadened the diagnosis to include women showing any two of three features: clinical and/or biochemical signs of hyperandrogenemia, ovulatory dysfunction, and polycystic ovarian morphology. In 2006, the Androgen Excess Society (AES) introduced a third set of criteria, highlighting the disorder’s variability. They suggested that diagnosis should be based on the presence of clinical or biochemical hyperandrogenism combined with ovulatory dysfunction, which includes ovulatory issues or polycystic ovarian morphology [1,2].

Current clinical guidelines acknowledge that women with PMOS face a markedly higher risk of MASLD due to IR, obesity, and hyperandrogenism. While MASLD screening in the general population remains controversial, studies indicate that women with PMOS, particularly those with additional metabolic risk factors, should be prioritized for assessment [48,49]. Initial screening includes abdominal ultrasound and liver function tests, specifically alanine aminotransferase (ALT) and aspartate aminotransferase (AST) [23,50]. For patients at higher risk of MASLD, such as those with obesity, prediabetes, and T2DM (many women with PMOS fall into this category), it is advised to bypass the ultrasound and directly assess fibrosis risk using the Fibrosis-4 index (FIB-4) to confirm steatosis [50,51]. If the FIB-4 score is >1.3, additional risk stratification is needed, which can include Vibration-Controlled Transient Elastography (VCTE) and/or Enhanced Liver Fibrosis (ELF) tests [51]. Individuals with a FIB-4 score greater than 1.3 and a VCTE-derived liver stiffness measurement (LSM) of 8.0 kPa or higher, or an ELF score of 9.8 or above, should be referred to liver specialty clinics (gastroenterology or hepatology) for further assessment, treatment, and long-term monitoring [51]. Lifestyle modifications, such as diet, weight loss, and exercise, are the most appropriate initial treatment options for PMOS patients with MASLD [23].

## 4. Nutrition Interventions: Evidence-Based Strategies

Current treatments for PMOS and MASLD are usually limited and focus on symptom management rather than the disease’s overall management. Nutritional strategies are often employed to address this challenge. Currently, these approaches aim to reduce inflammation, IR, and body weight through low-glycemic, anti-inflammatory, and calorie-controlled diets. Key additional approaches include the DASH (Dietary Approaches to Stop Hypertension) and Mediterranean diets, increased intake of healthy fats, and dietary supplements such as inositols and vitamin D [52,53,54]. Achieving a 7–10% weight loss is crucial for managing both conditions [5].

The following section reviews evidence from roughly 30 scholarly articles on dietary patterns, macronutrient-focused dietary strategies, and dietary supplement interventions. It highlights outcomes relevant to both conditions individually and together. The articles analyzed were identified through searches on PubMed and Google Scholar conducted in March 2026. Selected articles involved adult participants with MASLD, PMOS, or both, were written in English, and focused on the effects of nutritional interventions on these diseases. Articles that used the previous nomenclature of NAFLD and PCOS were included.

### 4.1. Dietary Patterns

Whole dietary patterns are increasingly attracting research interest due to their potential to mitigate metabolic disorders, liver fat accumulation, and androgen-related overproduction issues. While no single diet has been definitively established as the most effective for treating PMOS or MASLD, several dietary patterns show promise. The three dietary patterns with the strongest evidence for women with PMOS or MASLD are the low glycemic index (GI) diet, the DASH diet, and the Mediterranean diet. The studies referenced in this section are summarized in Table 1.

#### 4.1.1. Low Glycemic Index Diets

The glycemic index (GI) and glycemic load (GL) are two dietary measures that show how carbohydrates affect the body. GI measures the impact of carbohydrate intake on blood glucose levels after eating, using a scale from 0 to 100. When glucose absorption is higher, blood sugar levels increase, resulting in a higher GI score. GL considers GI as well as the carbohydrate content per serving, providing a more precise, practical, and complete picture of food’s effect on blood sugar [63]. Low-GI diets focus on consuming low-GI foods such as green vegetables, most fruits, kidney beans, chickpeas, and lentils, while avoiding high-GI foods such as white rice, white bread, and potatoes [64].

Research indicates that women diagnosed with PMOS who adhere to a low-GI diet observe improvements in both clinical and biochemical characteristics of the condition (Table 1). Specifically, these improvements include a notable reduction in acne and hirsutism [55,65], enhanced menstrual cyclicity [55,56], decreased total testosterone levels, and elevated SHBG levels [55]. A recent systematic review and meta-analysis of 10 randomized controlled trials (RCTs) demonstrated that low-GI diets further improved glucose regulation (fasting insulin and Homeostatic Model Assessment of Insulin Resistance, HOMA-IR), lipid profiles (TC and TGs), and waist circumference (WC) compared with high-GI diets [66].

#### 4.1.2. The DASH Diet

The DASH diet is a flexible, heart-healthy dietary pattern that was initially developed to reduce blood pressure and cholesterol levels by decreasing sodium intake and increasing intake of potassium, calcium, magnesium, fiber, and protein. The eating plan focuses on increasing the consumption of vegetables, fruits, and whole grains while limiting foods high in saturated and trans fats [67].

The DASH diet has been extensively studied for its beneficial effects on obesity, MetS, T2DM, CVD, and depression [68,69]; however, research on its effects on MASLD and/or PMOS remains limited. As shown in Table 1, women with PMOS who adhered to the DASH diet over a 2–3-month period exhibited reductions in body mass index (BMI), TG, and LDL-C levels, as well as improvements in antioxidant markers [58,59]. A recent double-blind RCT in 40 patients with obesity and MASLD showed decreases in body weight, hemoglobin A1c (HbA1C), AST, LPS, and inflammatory biomarkers after 8 weeks on the DASH diet [57]. These studies collectively indicate the potential of the DASH diet to improve markers associated with PMOS and MASLD.

#### 4.1.3. The Mediterranean Diet

The term “Mediterranean diet” generally refers to the traditional eating patterns of people in the sixteen countries bordering the Mediterranean Sea. Often recognized as one of the healthiest diets by health organizations and dietitians, the Mediterranean diet (Med Diet) focuses on vegetables, whole grains, nuts, legumes, and olive oil, with moderate amounts of fish and seafood. Refined carbohydrates, red or processed meats, sugars, sugary drinks, and saturated fats are limited [70]. The Med Diet has been extensively studied for its protective effects on cardiovascular health [71]. While individual components of the Med Diet have been examined in relation to PMOS and MASLD, research on the diet as a whole remains limited (see Table 1).

A recent study investigated adherence levels to the Med Diet among women with and without PMOS, focusing on hyperandrogenemia, inflammatory status, and IR. The findings showed that women with PMOS were generally less likely to follow the Med Diet compared to women without the condition. Additionally, the study found that factors such as inflammatory markers, monounsaturated fatty acid intake, and dietary adherence had the greatest impact on testosterone levels [61]. This is supported by previous studies showing that Med Diet adherence is inversely associated with IR [72], adiposity [73], and the risk of CVD [74] and T2DM [75]. Adherence to the Mediterranean Diet has also been associated with benefits for MASLD. A recent cohort study involving 2288 adults without hepatic steatosis, monitored over an average period of 5.3 years, indicated that individuals adhering to the Med Diet experienced the most significant reductions in hepatic steatosis [60]. Furthermore, a six-week RCT observed increases in insulin sensitivity and decreases in hepatic steatosis in participants following the Med Diet compared to those on a control low-fat, high-carbohydrate diet [62].

### 4.2. Macronutrient-Focused Dietary Strategies

In addition to overall dietary patterns, adjusting specific macronutrients can help address the shared underlying mechanisms of PMOS and MASLD. There is evidence supporting modifications such as reducing carbohydrates, choosing specific protein sources and amounts, and altering the types of dietary fats. The studies referenced in this section are summarized in Table 2.

#### 4.2.1. Carbohydrate Restriction

Carbohydrate-restricted diets (CRDs) are commonly employed to facilitate weight loss by diminishing appetite through the production of ketone bodies, thereby enhancing energy expenditure and insulin sensitivity, and promoting lipolysis. A typical CRD limits carbohydrate intake to under 45% of total calories. There are three main types of CRD: very-low-carbohydrate (VLCD) or ketogenic diets (≤10% of calories or 20–50 g/day), low-carbohydrate diets (LCD, 10–26% of calories or 50–130 g/day), and moderate carbohydrate diets (MCD, 26–45% of calories or 130–230 g/day) [88]. Several studies have been conducted regarding the advantages of CRDs for women with PMOS and/or MASLD. Here, we review several of these studies, with their findings summarized in Table 2.

A recent retrospective study investigated the effects of a very-low-calorie ketogenic diet on markers associated with metabolic and ovulatory dysfunction. After 12 weeks, women with concurrent PMOS and obesity exhibited reductions in BMI, waist circumference, and HOMA-IR. Additionally, decreases in anti-Müllerian hormone (AMH) and increases in progesterone and SHBG levels were observed [76]. These findings are supported by a 24-week pilot study that examined the effects of a VLCD (<20 g carbohydrates per day) on women with PMOS and obesity. In that study, the women experienced greater reductions in body weight and fasting insulin, along with lower free testosterone and Luteinizing Hormone (LH)/Follicle-Stimulating Hormone (FSH) ratio [78].

Even a moderate reduction in carbohydrate intake has been shown to benefit women diagnosed with PMOS. An eight-week crossover study revealed that when women with PMOS decreased their carbohydrate intake to 41% of total calories (compared to 55% in the eucaloric control), they experienced reductions in insulin area under the curve (AUC) and changes in body composition. These changes included decreases in fat mass in subcutaneous abdominal, intra-abdominal, and thigh intermuscular adipose tissues. Lean mass was preserved on the MCD but not in the control diet [77].

#### 4.2.2. Protein Quantity and Source

Proteins are essential macromolecules that play key roles in metabolic processes and overall health. They help reduce liver fat, increase satiety, improve insulin sensitivity, and support the maintenance of muscle mass. Together, these effects promote effective weight loss and management [89], which are important in treating both PMOS and MASLD (see Table 2).

Sources of dietary protein include both plant and animal foods. While each offers distinct advantages, plant-derived proteins have demonstrated superior metabolic benefits, whereas animal-derived proteins are more frequently linked to adverse effects. A recent case–control study involving 243 adults (including 121 individuals with MASLD) found that those who consumed more plant-derived proteins—such as vegetables, nuts, and grains—had a significantly reduced risk of MASLD. Conversely, higher consumption of meat-based proteins was associated with an increased risk of MASLD and greater hepatic fat accumulation [79]. Limited research has explored the benefits of plant-based proteins for women diagnosed with PMOS. A prospective, quasi-randomized, double-blind, placebo-controlled study involving 137 women with PMOS demonstrated that after three months of genistein consumption—a phytoestrogenic isoflavone mainly found in soy products—the participants exhibited decreased levels of LH, dehydroepiandrosterone sulfate (DHEAS), and testosterone. Furthermore, they demonstrated lower LDL-C and TG levels than the control group [82].

Overall, higher total protein intake has been significantly associated with a lower risk of MASLD, even after adjustment for multiple confounding factors [79]. This is further demonstrated to be true for women with PMOS. A single-blind RCT involving 33 women with PMOS demonstrated that a hypocaloric, protein-supplemented diet (containing 240 kcal of whey protein) resulted in greater reductions in weight and fat mass. Additionally, it was associated with lower serum cholesterol and apoprotein B levels compared with a diet supplemented with simple sugars. It is noteworthy, however, that HDL-C levels also declined in the protein-supplemented diet relative to the simple sugar-supplemented diet. There were no changes in sex steroids or SHBG as a result of the intervention [80].

Whey protein (WP), used as a nutritional supplement, has been investigated for its role in managing glycemic regulation in individuals with T2DM, thereby making it a potential treatment option for women diagnosed with PMOS. Studies involving patients with T2DM have demonstrated that WP intake can help maintain blood glucose levels, augment insulin secretion [90,91], and improve the incretin response [90]. A small exploratory study involving 14 women with PMOS and 15 healthy controls examined the short-term effects of 35 g of WP supplementation. The results showed that after just 7 days of WP intake, women with PMOS exhibited suppressed glucose levels relative to their baseline. This was linked to higher circulating levels of insulin and glucagon. Although no significant changes were observed in SHBG, TG, ALT, or AST due to WP supplementation, there was a slight decline in the AST:ALT ratio over time. Additionally, although not statistically significant, total cholesterol levels dropped by about 13% in women with PMOS after 7 days of WP intake [81].

#### 4.2.3. Dietary Fat Quality

Research has shown that the type and amount of dietary fats consumed can greatly affect health outcomes and benefits [92,93,94]. Dietary fats, also called lipids or fatty acids, are grouped into three main types: saturated fatty acids (SFA), monounsaturated fatty acids (MUFA), and polyunsaturated fatty acids (PUFA). This classification depends on the number and presence of double bonds in the fatty acid molecule: SFAs have no double bonds, MUFAs (also known as omega-9s, *n*-9) have one double bond, and PUFAs have two or more double bonds. PUFAs are further divided into omega-3 (*n*-3) and omega-6 (*n*-6) families, based on the location of the first double bond from the methyl end.

Both *n*-3 and *n*-6 PUFAs are regarded as essential nutrients, as humans and animals lack the enzymatic capacity to synthesize them. The predominant source of long-chain *n*-3 PUFAs is marine phytoplankton, which constitutes a vital component of the marine food chain [95]. Linoleic acid (C18:2*n*-6, LA) and α-linolenic acid (C18:3*n*-3, ALA) serve as precursors for *n*-6 and *n*-3 long-chain PUFAs, respectively. These acids compete for identical enzymes during the metabolic conversion of their derivatives. This is particularly significant given that long-chain *n*-6 PUFAs, such as arachidonic acid (C20:4*n*-6, AA), are believed to promote inflammation. In contrast, long-chain *n*-3 PUFAs, such as eicosapentaenoic acid (20:5*n*-3, EPA) and docosahexaenoic acid (22:6*n*-3, DHA), are considered anti-inflammatory.

A recent randomized controlled trial examined the effects of canola oil (high in *n*-3 PUFAs and MUFAs) and olive oil (rich in MUFAs and dietary polyphenols) compared to sunflower oil (high in *n*-6 PUFAs) in women with PMOS. After a 10-week intervention, participants who used canola oil showed reductions in TG, TC/HDL ratio, LDL/HDL ratio, and TG/HDL ratio. No significant changes in the lipid profile were seen with olive oil intake. Both oils led to decreases in fatty liver grade and HOMA-IR levels [83]. These results differ from another study that compared the effects of canola and olive oil in Asian Indian men with MASLD. Participants used either olive oil, canola oil, or a common soybean/safflower oil as a cooking medium (not to exceed 20 g/day) for 6 months. Results showed that the olive oil group experienced significant reductions in body weight, BMI, fasting insulin, fasting blood glucose, and other markers of IR, while HDL-C levels increased. Canola oil was associated with decreases in fasting glucose and TG. Both oils contributed significantly to a reduction in fatty liver severity grading [84]. The difference observed between the study involving women diagnosed with PMOS and men with MASLD could be partly due to variations in disease progression stages, studied populations, age groups, and intervention durations.

Aside from their anti-inflammatory properties, long-chain *n*-3 PUFAs, notably EPA and DHA, are recognized for their cardioprotective effects and their capacity to lower TG levels [96,97], making them ideal candidates for nutritional interventions in the treatment of PMOS and MASLD. A randomized crossover study involving women with PMOS and obesity showed that after 8 weeks, women taking 4 g/day of DHA + EPA experienced significant reductions in liver fat, TG, systolic blood pressure (BP), and diastolic BP compared to placebo. There were no significant changes in SHBG, free androgen index (FAI), or testosterone [86]. More recently, a double-blind RCT was conducted to evaluate the effect of *n*-3 supplementation (180 g EPA and 120 mg DHA) on PMOS symptoms and MetS. After 6 months, the 88 women with PMOS experienced increased HDL-C and decreased WC, LDL-C, TG, and TC compared with the control group. Furthermore, the women given *n*-3 had shorter menstrual cycle intervals at the end of the study than the control group [87].

Regarding the effects of *n*-3 PUFAs on MASLD, a double-blind, placebo-controlled, randomized clinical trial was conducted involving 103 adults diagnosed with MASLD, in which participants consumed 4 g of an EPA + DHA supplement daily for 15 to 18 months [85]. Upon conclusion of the study, the researchers observed a slight trend towards a reduction in hepatic fat; however, this finding was not statistically significant. Nevertheless, a secondary analysis demonstrated that erythrocyte DHA enrichment—a marker of adherence—was linearly correlated with a decreased fat percentage. No significant alterations were noted in liver enzyme levels [85].

Finally, research on the effect of *n*-3 PUFAs on IR has produced inconsistent results. Some studies show metabolic improvements in patients with T2DM [98,99,100], while others report no significant effect of these fatty acids or fish oil supplements [101,102,103]. A recent meta-analysis of 10 RCTs examining omega-3 supplementation in women with PMOS found that omega-3s improved insulin levels and HOMA-IR but did not significantly affect serum glucose [104]. This conflicting evidence might partly result from differences in disease types, age groups, populations studied, and lengths of interventions.

### 4.3. Dietary Supplement Interventions

Numerous micronutrients and bioactive compounds have been studied in relation to PMOS, MASLD, and both. This suggests that deficiencies or supplements could affect common pathophysiological pathways such as insulin signaling, oxidative stress, and liver lipid metabolism. In this section, we review three such compounds: vitamin D, inositol, and vitamin E, with their studies summarized in Table 3.

#### 4.3.1. Vitamin D

Vitamin D (VD) is a fat-soluble vitamin that can be synthesized endogenously or obtained through dietary sources from both animal and plant origins [114]. Although VD supplementation has been utilized to manage T2DM, leading to improved glycemic control and insulin response [115], there is limited data regarding its impact on PMOS and MASLD.

A double-blind, randomized, placebo-controlled study conducted in the United Kingdom involved 37 women with PMOS who took a high dose of VD (3200 IU) daily for three months. The researchers observed that, compared to a placebo, VD supplementation resulted in a modest decrease in ALT. Within-group comparisons of the VD group revealed that ALT, hyaluronic acid, and the ELF score all decreased after three months [105]. This minor effect of VD on ALT levels was also observed in another randomized, double-blind, placebo-controlled trial. The study, conducted in Iran with 53 adult participants diagnosed with MASLD, involved administering 50,000 IU of VD once every 14 days over four months. This intervention resulted in only a slight decrease in ALT levels, which was not statistically significant. Nonetheless, the researchers reported significant reductions in malondialdehyde (MDA), a marker of lipid peroxidation, and nearly significant reductions in high-sensitive C-reactive protein (hs-CRP) [106]. Further research is required to elucidate the effects of VD on PMOS and MASLD.

#### 4.3.2. Inositol (Myo-Inositol/D-Chiro-Inositol)

Inositols are polyols characterized by a six-carbon ring structure, with each carbon atom hydroxylated. Several of these sugar alcohol isomers exhibit biological activity, among which myo-inositol (MI) and D-chiro-inositol (DCI) are the most prevalent and are frequently recommended for the treatment of PMOS symptoms [116,117]. Furthermore, emerging research indicates that MI and DCI may also prove beneficial in the treatment of MASLD.

As previously mentioned, MI and DCI are often recommended to women for managing PMOS symptoms. However, studies have indicated that DCI supplementation alone might be ineffective (and potentially harmful) in women with PMOS, as they tend to have higher levels of the compound already [108,117]. A randomized, interventional, open-label study was conducted in 2019 to test seven different MI/DCI supplementation ratios in women with PMOS. The women were instructed to ingest 2 g per day of their assigned MI-to-DCI ratio for three months. At the end of the study, the researchers found that all seven ratios led to decreases in HOMA-IR and insulin levels. Furthermore, it was shown that the 40:1 MI-to-DCI ratio most effectively restored ovulation by decreasing LH and increasing progesterone, SHBG, and estradiol (E2) levels. None of the seven ratios tested had a significant effect on FSH levels [108]. These findings are further supported by a recent prospective clinical study involving 90 women with PMOS who took 1 g of MI twice daily for six months. By the end of the intervention, researchers found that 68% of the women experienced restored menstrual cycle regularity and had lower LH and LH/FSH ratios. They also noted reductions in fasting insulin and HOMA-IR [107].

Regarding the impact of MI on MASLD, two scholarly articles published in 2023 by the same research group indicated that MI may also benefit individuals with obesity and MASLD. These studies used a double-blind, placebo-controlled, randomized controlled trial design and involved 48 participants who ingested 4 g/day of MI for 8 weeks [109,110]. While both studies examined liver function, the first study also documented changes in cardiometabolic factors and anthropometric measures, whereas the second focused on inflammatory responses. Overall, the first study showed that 8 weeks of MI supplementation in people with MASLD and obesity led to lower body weight and systolic BP. They also observed decreases in fasting insulin, fasting glucose, HbA1C, and IR [110]. Regarding inflammation, the second study demonstrated that MI supplementation reduced TNF-α mRNA expression compared with placebo. They also observed decreases in the systemic inflammation response index and the monocytes-to-lymphocyte ratio in the MI group [109]. Both studies showed reductions in ALT, AST, and TC levels after MI supplementation. Additionally, they found that one in three participants experienced a one-grade reduction in MASLD severity as a result of the intervention [109,110].

#### 4.3.3. Vitamin E

Vitamin E constitutes a group of fat-soluble compounds, predominantly alpha-tocopherol, which function as essential antioxidants by protecting bodily tissues from damage caused by free radicals. Dietary sources rich in vitamin E include vegetable oils such as canola and olive oils, as well as nuts and seeds. Furthermore, meats, dairy products, leafy greens, and fortified cereals are also significant sources of vitamin E [118,119,120].

Vitamin E has been shown to improve the histological characteristics of MASH in both preclinical and clinical research. Multiple clinical practice guidelines endorse the use of vitamin E as an off-label therapeutic option for MASH [121]. Indeed, the American Association for the Study of Liver Disease (AASLD) includes vitamin E among the potential pharmacological treatments for MASLD [122]. This is supported by an RCT involving 247 adults with MASH without T2DM that compared 800 IU/day of Vitamin E to pioglitazone over 96 weeks. At the end of the intervention, researchers observed histological evidence of reduced hepatic inflammation and steatosis, along with lower ALT and AST levels in the vitamin E group. The fibrosis score did not change with either vitamin E or pioglitazone [113].

Regarding the relationship between PMOS and Vitamin E, more research is still necessary. Two studies from the same group, published in 2019, showed that when Vitamin E was co-supplemented with CoQ10 in 86 women with PMOS, fasting glucose, HOMA-IR, TC, LDL-C, and diastolic BP decreased. Additionally, HDL-C and SHBG increased in the vitamin E + CoQ10 group [111,112]. This is supported by a systematic review and meta-analysis that demonstrated vitamin E’s significant improvement of lipid profiles and reduction in insulin levels and HOMA-IR. The review further indicated that, while vitamin E decreases LH and testosterone levels, it increases FSH and progesterone concentrations [123].

## 5. Discussion and Future Directions

The purpose of this narrative review was to examine the pathophysiological link between PMOS and MASLD and evaluate the effectiveness of specific nutritional interventions. The connection between PMOS and MASLD emphasizes the importance of a comprehensive approach to these conditions and highlights the need to address the underlying metabolic disturbances that contribute to both. Notwithstanding this association, it is imperative to note that the evidence presented in this narrative review predominantly originates from discrete studies—specifically focusing on PMOS-only and MASLD-only populations—rather than from cohorts that exhibit both conditions.

We identified that adherence to dietary patterns like the Mediterranean and DASH diets currently has strong evidence of therapeutic benefits for PMOS and MASLD. Additionally, we showed that changes in dietary lipids, particularly increased intake of *n*-3 PUFA, have robust evidence supporting their benefits for both conditions among the macronutrients. This finding was uniquely supported by a PMOS trial that used liver fat as a primary outcome [86]. Among dietary supplements, MI has the strongest evidence for improving IR and ovulation in PMOS, although evidence on hepatic effects remains limited. Vitamin E is another promising candidate, especially given its strong support as a treatment for MASLD. Further research is necessary, however, to understand its effects on women with PMOS.

### 5.1. Current Gaps and Limitations

A notable gap in current research is the lack of RCTs specifically focusing on women with both PMOS and MASLD. Most studies on nutritional interventions are carried out either on PMOS or MASLD alone. This is concerning because women with both conditions may form a unique subgroup—characterized by higher IR, increased hepatic fat, and more severe androgen excess—that could respond differently to treatments compared to women with only one condition. Additionally, since the risk of MASLD is significantly higher among women with PMOS (and vice versa), there is a greater need for targeted screening and management approaches to reduce this risk.

Adding to the challenge is the lack of consensus on diagnosing PMOS. While the Rotterdam Criteria are most used for PMOS diagnosis, several articles examined in this paper employed different criteria, making direct comparison difficult since each creates different phenotypic groups [1,2]. Additionally, due to inconsistent diagnostic standards and limited awareness, women with PMOS often experience a lengthy delay before receiving a definitive diagnosis, frequently consulting multiple healthcare providers. A global cross-sectional study involving 1385 women showed that more than one-third waited over two years and saw at least three healthcare professionals before obtaining an official diagnosis [124,125]. Part of the delay in diagnosis was believed to stem from confusion caused by the previous name of polycystic ovary syndrome (PCOS). The old name failed to reflect the disorder’s multisystem nature and implied pathological ovarian cysts. As a result, earlier this year, it was announced that PCOS would be renamed Polyendocrine Metabolic Ovarian Syndrome (PMOS). This change aims to better represent the disease’s complexity, potentially leading to increased awareness, more accurate diagnosis, enhanced care, higher patient satisfaction, and better outcomes across the condition’s various features [126].

MASLD also presents diagnostic challenges. Diagnosis can be made through various methods, including ultrasound, magnetic resonance elastography, proton density fat fraction (MRE-PDFF), liver biopsy, or enzyme surrogates such as ALT and AST [127,128]. Each method has varying sensitivities, clinical implications, and drawbacks. Additionally, in 2023, it was decided that nonalcoholic fatty liver disease (NAFLD) should be renamed as Metabolic Dysfunction-Associated Steatotic Liver Disease (MASLD). While this change aims to better reflect the disease’s underlying cause and promote non-stigmatizing language, it also makes comparisons with earlier studies more challenging [7]. Many of the articles reviewed for this paper used the older nomenclatures of NAFLD or PCOS.

Multiple methodological limitations are evident across the analyzed studies. For instance, intervention durations ranged from 1 week to 5.3 years, with many lasting between 8 and 12 weeks. This period is insufficient to accurately evaluate the progression of hepatic fibrosis, which may span several years [129]. Furthermore, there was a clear lack of demographic diversity among the study participants. A large portion of these studies mainly focused on white women from high-income countries. Meanwhile, Black, Hispanic, and Indigenous women remain significantly underrepresented despite having unique hormonal, metabolic, and socioeconomic risk profiles for both PMOS [130] and MASLD [131]. Overall, longer nutritional intervention studies that include more diverse populations are needed in PMOS and MASLD research.

### 5.2. Emerging Areas and Future Research

There are several emerging areas of nutrition research that warrant investigation regarding their potential impact on PMOS and MASLD. These include time-restricted eating (TRE) or intermittent fasting, precision nutrition, and interventions targeting the gut microbiome. TRE is a dietary intervention where all nutritional intake occurs within a 6- to 11 h window, without changing diet quality or quantity. Clinical TRE trials with a 6- to 10 h eating window have reported various health benefits, including weight loss, improved glucose regulation, lower BP, and improved cholesterol [132]. A recent systematic review investigated how TRF affects fertility and reproductive hormones in women with PMOS. It found that TRF significantly improved menstrual regularity, insulin sensitivity, and hyperandrogenism [133]. However, it is important to note that the review included only three articles, emphasizing the need for further research in this area.

Precision Nutrition (PN) is a methodology for devising personalized and adaptable nutritional guidance that considers individual factors such as genetics, microbiome, metabolic profile, health status, physical activity, dietary habits, food environment, as well as socioeconomic and psychosocial characteristics [134]. Conceptually, PN represents a promising approach for phenotypically diverse diseases such as PMOS and MASLD; however, it remains in its early stages, with numerous tools still in development. A recent RCT involving 97 adults with MASLD and obesity examined whether inflammatory markers could be used as diagnostic tools and guides for personalized nutrition in MASLD. They found that leptin and Leukocyte cell-derived chemotaxin-2 (LECT2) could not only effectively diagnose the disease throughout the intervention but also help develop a predictive score for greater weight loss, aiding in choosing the best strategy for MASLD treatment [135].

As previously mentioned, emerging research suggests that the gut microbiome may contribute to metabolic disorders such as PMOS and MASLD. Therefore, dietary fiber, fermented foods, probiotics, and synbiotics might affect microbial composition beyond just macronutrients. A systematic review and meta-analysis of thirteen studies involving women with PMOS examined the effects of probiotics, prebiotics, and synbiotics on hormonal and inflammatory biomarkers. The results showed that administering synbiotics and probiotics led to improvements in hormonal parameters—such as the FAI and SHBG—as well as inflammatory markers, including nitric oxide and MDA [136].

Further research is crucial to enhance our understanding of the connection between PMOS and MASLD, especially regarding pathophysiology, genetics, and effective nutritional strategies. Although studies have identified specific genetic markers linked to both conditions, like the PNPLA3 gene variant in MASLD [9] and various polymorphisms in PMOS [3], there remains a need for comprehensive genetic and epigenetic research. Such studies should aim to explore the full range of genetic interactions and epigenetic changes that contribute to both disorders, potentially uncovering shared genetic pathways that could be targeted with treatments. Additionally, long-term longitudinal studies are necessary to monitor patients with PMOS and/or MASLD over time to better understand disease progression and natural history. Furthermore, interventional research examining the effectiveness of various treatments—including lifestyle changes, medications, and surgeries—could offer valuable insights into managing patients with both conditions. Addressing these areas will significantly advance our understanding of PMOS and MASLD, leading to improved diagnostic methods, more effective treatments, and ultimately better patient outcomes.

## 6. Clinical Implications and Conclusions

The coexistence of PMOS and MASLD increases the risk of metabolic complications, such as T2DM and CVD. Therefore, clinicians managing patients with PMOS should be alert for signs of MASLD and vice versa. Early detection and treatment of MASLD in PMOS patients are essential because these women can develop MASLD at a relatively young age. Prompt identification and management of these risk factors are vital in preventing the progression of both conditions and improving overall patient outcomes. This approach not only enhances clinical results but also improves the quality of life for affected women.

Both PMOS and MASLD greatly benefit from lifestyle changes, including dietary improvements, weight management, and increased physical activity. These actions can help reduce IR, improve lipid profiles, decrease liver fat buildup, and ease symptoms of hyperandrogenism in PMOS [3]. Based on the findings discussed in this paper, some practical recommendations for clinicians and registered dietitian nutritionists (RDNs) working with these populations include first recommending an anti-inflammatory dietary pattern, such as the Mediterranean Diet or the DASH Diet, as a framework. Next, the focus may shift to increasing their intake of healthier fats, such as *n*-3 PUFAs (through food or supplements) and myo-inositol (4 g/day MI or 40:1 MI/DCI combination), since both have been shown to benefit women with PMOS and MASLD. Lastly, vitamin D levels could be evaluated and supplementation provided for those with a deficiency. It should be noted that although these strategies are among the most biologically plausible and clinically feasible options from the reviewed literature, many still need direct testing in women with coexisting PMOS and MASLD. Dietary interventions should remain the main approach for managing women with PMOS and/or MASLD, but more advanced cases might require medications like Metformin, which addresses IR and metabolic problems, or specific liver treatments such as antioxidants and anti-inflammatory agents [137].

## 7. Conclusions

In conclusion, the literature highlights the intricate connection between PMOS and MASLD. Both are associated with metabolic dysfunction and shared risk factors such as IR, obesity, and hyperandrogenism. Despite this connection, the evidence presented in this review was drawn from separate literature (PMOS-only and MASLD-only) rather than from populations with both conditions. Future research should involve longer-term RCTs focusing on women from diverse backgrounds who have been diagnosed with both PMOS and MASLD. These conditions, either alone or together, pose an increasing burden among women of reproductive age worldwide. Studying these populations can not only have a significant clinical impact but also advance overall public health.

## Figures and Tables

**Table 1:** Summary of Research on Dietary Patterns as Nutritional Interventions for PMOS and MASLD.

Intervention	Study Type	Population (Diagnosis Criteria)	N	Duration	PMOS- Specific Outcomes	MASLD/ Metabolic Outcomes	Ref
Low-GI Diet	RCT	Women with obesity or overweight (PMOS = Rotterdam Criteria)	62 Total PMOS = 28; non-PMOS = 34	24 wk.	↓ testosterone, FAI, and acne ↑SHBG and menstrual regularity	↔ HOMA-IR and weight loss	[55]
Low-GI Diet	RCT	Women with PMOS (Rotterdam Criteria)	96	12 mo.	↑ menstrual regularity ↔ androgenic hormones	↑ insulin sensitivity, hemostasis ↔ blood lipids	[56]
DASH Diet	double-blind RCT	Patients with obesity and MASLD (confirmed by ultrasonography)	40 Total DASH = 20 (13 female); Control = 20 (12 female)	8 wk.	Not assessed	↑ weight loss ↓ HbA1c, AST, LPS, and inflammatory biomarkers	[57]
DASH Diet	RCT	Women with PMOS (Rotterdam criteria)	48 Total DASH = 24; Control = 24	8 wk.	Not assessed	↓ BMI, TG, and LDL-C ↑ weight loss, antioxidant capacity, and GSH	[58]
DASH Diet	RCT	Women with PMOS (Rotterdam criteria)	60 Total DASH = 30; Control = 30	3 mo.	↑ SHBG ↓ androstenedione	↑ weight loss, and antioxidant status ↓ BMI, and fat mass	[59]
Mediterranean Diet	Cohort study	Adults without hepatic steatosis (FLI and NAFLD score)	2288 total (65.4% women)	Mean 5.3 yr follow-up	Not assessed	↓ hepatic steatosis with adherence to the Med Diet	[60]
Mediterranean Diet	Case–control, cross-sectional study	Women of reproductive age (PMOS = Rotterdam criteria)	224 Total PMOS = 112; Control = 112		↓ Adherence to Med Diet in PMOS Testosterone negatively correlated with Med Diet score	Not directly assessed	[61]
Mediterranean Diet	Randomized crossover	Non-diabetic adults with MASLD (biopsy-proven)	12 Total (6 females)	6 wk.	Not assessed	↑ insulin sensitivity ↓ hepatic steatosis ↔ weight loss	[62]

↑ = increased; ↓ = decreased; ↔ = no change; AST = aspartate aminotransferase; BMI = body mass index; DASH = Dietary Approaches to Stop Hypertension; FAI = free androgen index; FLI = Fatty Liver Index; GI = Glycemic Index; GSH = Glutathione; HbA1c = hemoglobin A1cC; HOMA-IR = Homeostatic Model Assessment of Insulin Resistance; LDL-C = low-density lipoprotein cholesterol; LPS = lipopolysaccharide; MASLD = Metabolic Dysfunction-Associated Steatotic Liver Disease; Med Diet = Mediterranean diet; NAFLD = non-alcoholic fatty liver disease; PMOS = Polyendocrine Metabolic Ovarian Syndrome; RCT = Randomized Controlled Trial; SHBG = sex hormone-binding globulin; TG = triglycerides.

**Table 2:** Summary of Research on Macronutrient-Focused Dietary Strategies as Nutritional Interventions for PMOS and MASLD.

Intervention	Study Type	Population (Diagnosis Criteria)	N	Duration	PMOS- Specific Outcomes	MASLD/ Metabolic Outcomes	Ref
Very-low-calorie ketogenic diet (weeks 1–4: 600–800 kcal/day; weeks 5–8: 1200–1500 kcal/day; weeks 9–12: 1500–2000 kcal/day)	retrospective study	Women with PMOS and Obesity (Rotterdam criteria)	25	12 wk.	↓ AMH ↑ progesterone and SHBG	↓ BMI, WC, and HOMA-IR index	[76]
Moderate carbohydrate diet (41% CHO) vs. eucaloric control (55% CHO) diet	crossover-diet intervention	Women with PMOS (NIH criteria)	30	8 wk.	Not assessed	↓ insulin AUC ↓ fat mass in subcutaneous-abdominal, intra-abdominal, and thigh-intermuscular adipose tissue	[77]
Very-low-carbohydrate, ketogenic diet (<20 g CHO/day)	pilot study	Women with PMOS (history of chronic anovulation and/or hyperandrogenemia) and Obesity or overweight (BMI ≥ 27 kg/m^2^)	11 (5 completed)	24 wk.	↓ free testosterone, LH/FSH ratio	↓ BW, fasting insulin	[78]
Plant vs. Animal Protein Sources	case–control study	Adults with and without MASLD (liver enzymes and ultrasound sonography)	243 Total MASLD = 121 (65 female); Control = 122 (64 female)		Not assessed	Higher total protein intake ↓ MASLD risk Higher plant-based protein ↓ MASLD risk Higher animal-based protein ↑ MASLD risk and hepatic fat	[79]
High Protein vs. Simple Sugar Diet (240 kcal/day with 450 kcal deficit)	single-blinded RCT	Women with PMOS (NIH Criteria)	24 Total Protein = 11; Sugar = 13	2 mo.	↔ sex steroids, and SHBG	↑ weight loss and fat mass loss ↓ cholesterol, HDL and apoprotein B	[80]
Whey Protein (35 g daily)	exploratory study	Women of reproductive age (PMOS = Rotterdam Criteria)	29 Total PMOS = 14; Controls = 15	7 d.	↔ SHBG	↓ glucose ↑ glucagon, insulin ↔ TG, AST, ALT	[81]
Genistein Supplementation (18 mg twice daily)	prospective quasi-randomized	Women with PMOS (Rotterdam Criteria)	137 Total Genistein = 69; Control = 68	3 mo.	↔ FSH ↓ LH, DHEAS, and testosterone	↔ HDL ↓ LDL, TG	[82]
Canola and Olive Oil compared to Sunflower Oil (25 g daily)	RCT	Women with PMOS (Rotterdam Criteria)	72 Total (24 in each group)	10 wk.	↔ SHBG	Canola oil: ↓ TG, TC/HDL, LDL/HDL, and TG/HDL Canola and Olive: ↓ fatty liver grade and HOMA-IR	[83]
Canola and Olive Oil compared to soybean/safflower oil (≤20 g/day as cooking medium)	randomized, parallel, open-label	Asian Indian males with MASLD (abdominal ultrasonography)	93 Total Canola = 33; Olive = 30; Control = 30	6 mo.	Not assessed	Olive Oil: ↓ BW, BMI, fasting insulin, HOMA-IR, ↑HDL, Canola Oil: ↓ fasting glucose, TG Both: ↓ fatty liver grade	[84]
Omega-3 FA (EPA + DHA 4 g/day)	RCT	Adults with MASLD (histological confirmation or imaging evidence of liver fat)	95 Total DHA + EPA = 47 (26 female); Control = 48 (17 female)	15–18 mo.	Not assessed	DHA enrichment linearly associated with ↓ liver fat%,	[85]
Omega-3 FA (DHA + EPA 4 g/day)	RCT	Women with PMOS (hyperandrogenism and oligomenorrhea) and obesity or overweight (BMI > 25 kg/m^2^)	25	8 wk.	↔ SHBG. FAI, testosterone	↓ Liver fat, TG, systolic BP, and diastolic BP	[86]
Omega-3 FA (2 g/day) vs. Olive Oil	double-blind clinical trial	Women with PMOS (NIH Criteria)	88	6 mo.	↑menstrual regularity	↓ WC, LDL, TG, TC ↑ HDL	[87]

↑ = increased; ↓ = decreased; ↔ = no change; ALT = alanine aminotransferase; AMH = anti-Müllerian hormone; AST = aspartate aminotransferase; AUC = area under the curve; BMI = body mass index; BP = blood pressure; BW = body weight; CHO = carbohydrates; DHA = docosahexaenoic acid; DHEAS = Dehydroepiandrosterone sulfate; EPA = eicosapentaenoic acid; FAI = free androgen index; FSH = follicle-stimulating hormone; HDL = high-density lipoprotein; HOMA-IR = Homeostatic Model Assessment of Insulin Resistance; LDL = low-density lipoprotein; LH = luteinizing hormone; MASLD = Metabolic Dysfunction-Associated Steatotic Liver Disease; NIH = National Institutes of Health; PMOS = Polyendocrine Metabolic Ovarian Syndrome; RCT = Randomized Controlled Trial; SHBG = sex hormone-binding globulin; TC = total cholesterol; TG = triglycerides; WC = waist circumference.

**Table 3:** Summary of Research on Micronutrients and Bioactive Compounds as Nutritional Interventions for PMOS and MASLD.

Intervention	Study Type	Population (Diagnosis Criteria)	N	Duration	PMOS- Specific Outcomes	MASLD/ Metabolic Outcomes	Ref
Vitamin D (3200 IU/day)	RCT	Women with PMOS (Rotterdam Criteria)	37 Total Vitamin D = 18; Placebo = 19	3 mo.	↔ testosterone, SHBG, FAI	Compared to Placebo ↓ ALT (both modest effect) Within Vitamin D: ↓ ALT, hyaluronic acid, ELF Score	[105]
Vitamin D (50,000 IU once every 14 days)	RCT	Adults with MASLD (ultrasonography scan and increased ALT)	53 Total Vitamin D =27 (13 females); Placebo = 26 (13 female)	4 mo.	Not assessed	↓ MDA (marker of lipid peroxidation) Near-significant ↓ hs-CRP ↔ hepatic enzymes, HOMA-IR, fatty liver grades	[106]
Myo-Inositol (1 g twice daily)	Prospective Clinical Study	Women with PMOS (Rotterdam criteria)	90	6 mo.	Restored menstrual cycle regularity (68%) ↓ LH, LH/FSH ratio	↓ fasting insulin, HOMA-IR	[107]
Myo-inositol/D-chiro-inositol (2 g/day, 7 different ratios)	Randomized, interventional, open-labeled	Women with PMOS (Rotterdam Criteria)	56	3 mo.	All Ratios: ↔ FSH 40:1 MI/DCI: best restored ovulation ↓ LH ↑ progesterone, SHBG, E2 levels	All Ratios: ↓ HOMA-IR, insulin	[108]
Myo-inositol (4 g/day)	double-blind placebo-controlled RCT	Adults with MASLD (ultrasonography) and obesity (BMI 30–40 kg/m^2^)	48 Total Myo-inositol = 24 (13 females) Placebo = 24 (11 females)	8 wk.	Not assessed	↓ fat mass, TNF-α, systemic inflammation response index ↑ fat-free mass	[109]
Myo-inositol (4 g/day)	double-blind placebo-controlled RCT	Adults with MASLD (ultrasonography) and obesity (BMI 30–40 kg/m^2^)	48 Total Myo-inositol = 24 (13 females) Placebo = 24 (11 females)	8 wk.	Not assessed	↓ BW, systolic BP. Fasting insulin, fasting glucose, HbA1c, insulin resistance, ↓ ALT, AST, TC, 1 in 3 reduced one grade in the severity of MASLD	[110]
Vitamin E and/or CoQ10	double-blind placebo-controlled RCT	Women with PMOS (Rotterdam Criteria)	86 Toal CoQ10 = 22; Vitamin E = 22; CoQ10 + Vitamin E = 21; Placebo = 21	8 wk.	All: ↓ testosterone, LH CoQ10 + Vitamin E: ↑ SHBG	CoQ10 + Vitamin E: ↓ Fasting glucose, HOMA-IR	[111]
Vitamin E and/or CoQ10	double-blind placebo-controlled RCT	Women with PMOS (Rotterdam Criteria)	86 Total CoQ10 = 22; Vitamin E = 22; CoQ10 + Vitamin E = 21; Placebo = 21	8 wk.	Not assessed	CoQ10 + Vitamin E: ↓ TC, LDL-C, diastolic BP ↑HDL-C	[112]
Vitamin E (800 IU/day) vs. pioglitazone	RCT	Adults with MASH (NAFLD activity Score) without T2DM	247 Total Vitamin E = 84; pioglitazone = 80; placebo = 83	96 wk.	Not assessed	↓ Hepatic Inflammation and steatosis (histology) ↓ ALT, AST ↔ fibrosis score	[113]

↑ = increased; ↓ = decreased; ↔ = no change; ALT = alanine aminotransferase; AST = aspartate aminotransferase; BP = blood pressure; BW = body weight; CoQ10 = Coenzyme Q10; DCI = D-chiro-inositol; E2 = Estradiol; ELF = enhanced liver fibrosis; FAI = free androgen index; FSH = follicle-stimulating hormone; HbA1c = hemoglobin A1C; HDL = high-density lipoprotein; HOMA-IR = Homeostatic Model Assessment of Insulin Resistance; hs-CRP = high-sensitive C-reactive protein; IU = international units; LDL = low-density lipoprotein; LH = luteinizing hormone; MASLD = Metabolic Dysfunction-Associated Steatotic Liver Disease; MDA = malondialdehyde; MI = myo-inositol; NAFLD = nonalcoholic fatty liver disease; PMOS = Polyendocrine Metabolic Ovarian Syndrome; RCT = Randomized Controlled Trial; SHBG = sex hormone-binding globulin; T2DM = Type 2 diabetes mellitus; TC = total cholesterol.

## Data Availability

No new data were created or analyzed in this study. Data sharing is not applicable to this article.

## Abbreviations

The following abbreviations are used in this manuscript:

AA	arachidonic acid
ALT	alanine aminotransferase
AMH	anti-Müllerian hormone
AST	aspartate aminotransferase
AUC	area under the curve
BMI	body mass index
BP	blood pressure
BW	body weight
CHO	carbohydrates
CoQ10	Coenzyme Q10
CRD	carbohydrate-restricted diets
CRP	C-reactive protein
CVD	cardiovascular diseases
DASH	Dietary Approaches to Stop Hypertension
DCI	D-chiro-inositol
DHA	docosahexaenoic acid
DHEAS	dehydroepiandrosterone sulfate
E2	estradiol
ELF	enhanced liver fibrosis
EPA	eicosapentaenoic acid
FAI	free androgen index
FSH	follicle-stimulating hormone
GI	glycemic index
GL	glycemic load
GSH	glutathione
GWAS	genome-wide association study
HbA1C	hemoglobin A1c
HDL	high-density lipoprotein
HOMA-IR	Homeostatic Model Assessment of Insulin Resistance
hs-CRP	high-sensitive C-reactive protein
IR	insulin resistance
LCD	low-carbohydrate diets
LDL-C	low-density lipoprotein cholesterol
LECT2	leukocyte cell-derived chemotaxin-2
LH	luteinizing hormone
LPS	lipopolysaccharide
MASH	metabolic dysfunction-associated steatohepatitis
MASLD	Metabolic Dysfunction-Associated Steatotic Liver Disease
MCD	moderate carbohydrate diets
MDA	malondialdehyde
Med Diet	Mediterranean diet
MetS	Metabolic syndrome
MI	myo-inositol
MRE-PDFF	magnetic resonance elastography, proton density fat fraction
*n*-3	omega-3
*n*-6	omega-6
NAFLD	Non-Alcoholic Fatty Liver Disease
PCOS	Polycystic Ovary Syndrome
PMOS	Polyendocrine Metabolic Ovarian Syndrome
PN	Precision Nutrition
RCT	randomized controlled trial
RDN	registered dietitian nutritionists
SHBG	sex hormone-binding globulin
T2DM	type 2 diabetes mellitus
TC	total cholesterol
TG	triglycerides
TNFα	Tumor Necrosis Factor-alpha
TRE	time-restricted eating
VD	Vitamin D
VLCD	very-low-carbohydrate diet
WC	waist circumference
WP	whey protein

## References

[1] Lizneva D., Suturina L., Walker W., Brakta S., Gavrilova-Jordan L., Azziz R. (2016). Criteria, Prevalence, and Phenotypes of Polycystic Ovary Syndrome. Fertil. Steril..

[2] Azziz R., Carmina E., Chen Z., Dunaif A., Laven J.S.E., Legro R.S., Lizneva D., Natterson-Horowtiz B., Teede H.J., Yildiz B.O. (2016). Polycystic Ovary Syndrome. Nat. Rev. Dis. Primers.

[3] Siddiqui S., Mateen S., Ahmad R., Moin S. (2022). A Brief Insight into the Etiology, Genetics, and Immunology of Polycystic Ovarian Syndrome (PCOS). J. Assist. Reprod. Genet..

[4] Diagnostic Criteria for Polycystic Ovary Syndrome|Ugeskriftet.Dk. https://ugeskriftet.dk/videnskab/diagnostiske-kriterier-polycystisk-ovariesyndrom.

[5] Kelley C.E., Brown A.J., Diehl A.M., Setji T.L. (2014). Review of Nonalcoholic Fatty Liver Disease in Women with Polycystic Ovary Syndrome. World J. Gastroenetrol..

[6] Yang M., Khoukaz L., Qi X., Kimchi E.T., Staveley-O’carroll K.F., Li G. (2021). Diet and Gut Microbiota Interaction-Derived Metabolites and Intrahepatic Immune Response in NAFLD Development and Treatment. Biomedicines.

[7] Rinella M.E., Sookoian S. (2023). From NAFLD to MASLD: Updated Naming and Diagnosis Criteria for Fatty Liver Disease. J. Lipid Res..

[8] Bellentani S., Marino M. (2009). Epidemiology and Natural History of Non-Alcoholic Fatty Liver Disease (NAFLD). Ann. Hepatol..

[9] Byrne C.D., Targher G. (2015). NAFLD: A Multisystem Disease. J. Hepatol..

[10] Polyzos S.A., Goulis D.G., Kountouras J. (2015). Nonalcoholic Fatty Liver Disease and Polycystic Ovary Syndrome. Ann. Hepatol..

[11] Manzhalii E.H., Tatarchuk T.F., Tutchenko T.M., Kosei N.V., Mnevets R.O. (2023). Relationships between Nonalcoholic Fatty Liver Disease and Polycystic Ovary Syndrome. Reprod. Endocrinol..

[12] Paschou S.A., Polyzos S.A., Anagnostis P., Goulis D.G., Kanaka-Gantenbein C., Lambrinoudaki I., Georgopoulos N.A., Vryonidou A. (2019). Nonalcoholic Fatty Liver Disease in Women with Polycystic Ovary Syndrome. Endocrine.

[13] DeFronzo R.A., Tobin J.D., Andres R. (1979). Glucose Clamp Technique: A Method for Quantifying Insulin Secretion and Resistance. Am. J. Physiol.-Endocrinol. Metab..

[14] Dunaif A., Segal K.R., Futterweit W., Dobrjansky A. (1989). Profound Peripheral Insulin Resistance, Independent of Obesity, in Polycystic Ovary Syndrome. Diabetes.

[15] Gambineri A., Patton L., Altieri P., Pagotto U., Pizzi C., Manzoli L., Pasquali R. (2012). Polycystic Ovary Syndrome Is a Risk Factor for Type 2 Diabetes: Results from a Long-Term Prospective Study. Diabetes.

[16] Baptiste C.G., Battista M.C., Trottier A., Baillargeon J.P. (2010). Insulin and Hyperandrogenism in Women with Polycystic Ovary Syndrome. J. Steroid Biochem. Mol. Biol..

[17] Rosenfield R.L., Ehrmann D.A. (2016). The Pathogenesis of Polycystic Ovary Syndrome (PCOS): The Hypothesis of PCOS as Functional Ovarian Hyperandrogenism Revisited. Endocr. Rev..

[18] Bano G. (2013). Glucose Homeostasis, Obesity and Diabetes. Best Pract. Res. Clin. Obstet. Gynaecol..

[19] Karakas S.E., Kyoungmi K., Duleba A.J. (2010). Determinants of Impaired Fasting Glucose versus Glucose Intolerance in Polycystic Ovary Syndrome. Diabetes Care.

[20] Wu J., Yao X.Y., Shi R.X., Liu S.F., Wang X.Y. (2018). A Potential Link between Polycystic Ovary Syndrome and Non-Alcoholic Fatty Liver Disease: An Update Meta-Analysis. Reprod. Health.

[21] Chen M.J., Chiu H.M., Chen C.L., Yang W.S., Yang Y.S., Ho H.N. (2010). Hyperandrogenemia Is Independently Associated with Elevated Alanine Aminotransferase Activity in Young Women with Polycystic Ovary Syndrome. J. Clin. Endocrinol. Metab..

[22] González F. (2012). Inflammation in Polycystic Ovary Syndrome: Underpinning of Insulin Resistance and Ovarian Dysfunction. Steroids.

[23] Vassilatou E. (2014). Nonalcoholic Fatty Liver Disease and Polycystic Ovary Syndrome. World J. Gastroenterol..

[24] Zhang T., Gao H., Fan Y., Chen S., Li Y., Liu R., Li T., Yin C. (2023). Gut Microbiota Disorder Induces Liver Dysfunction in Polycystic Ovary Syndrome Rats’ Model by Regulating Metabolite Rosmarinic Acid. Life Sci..

[25] Kelley S.T., Skarra D.V., Rivera A.J., Thackray V.G. (2016). The Gut Microbiome Is Altered in a Letrozole-Induced Mouse Model of Polycystic Ovary Syndrome. PLoS ONE.

[26] Zhao X., Jiang Y., Xi H., Chen L., Feng X. (2020). Exploration of the Relationship Between Gut Microbiota and Polycystic Ovary Syndrome (PCOS): A Review. Geburtshilfe Frauenheilkd..

[27] Singh V., Mahra K., Jung D.R., Shin J.H. (2024). Gut Microbes in Polycystic Ovary Syndrome and Associated Comorbidities; Type 2 Diabetes, Non-Alcoholic Fatty Liver Disease (NAFLD), Cardiovascular Disease (CVD), and the Potential of Microbial Therapeutics. Probiotics Antimicrob. Proteins.

[28] Mei Y., Li W., Wang B., Chen Z., Wu X., Lin Y., Wang M. (2025). Gut Microbiota: An Emerging Target Connecting Polycystic Ovarian Syndrome and Insulin Resistance. Front. Cell. Infect. Microbiol..

[29] Sun Y., Gao S., Ye C., Zhao W. (2023). Gut Microbiota Dysbiosis in Polycystic Ovary Syndrome: Mechanisms of Progression and Clinical Applications. Front. Cell. Infect. Microbiol..

[30] Kassi E., Pervanidou P., Kaltsas G., Chrousos G. (2011). Metabolic Syndrome: Definitions and Controversies. BMC Med..

[31] Onat A. (2011). Metabolic Syndrome: Nature, Therapeutic Solutions and Options. Expert Opin. Pharmacother..

[32] Lavor C.B.H., Viana A.B., Medeiros F.D.C. (2022). Polycystic Ovary Syndrome and Metabolic Syndrome: Clinical and Laboratory Findings and Non-Alcoholic Fatty Liver Disease Assessed by Elastography. RBGO Gynecol. Obstet..

[33] Giri A., Joshi A., Shrestha S., Chaudhary A. (2022). Metabolic Syndrome among Patients with Polycystic Ovarian Syndrome Presenting to a Tertiary Care Hospital: A Descriptive Cross-Sectional Study. J. Nepal. Med. Assoc..

[34] Zahiri Z., Sharami S.H., Milani F., Mohammadi F., Kazemnejad E., Ebrahimi H., Dalil Heirati S.F. (2015). Metabolic Syndrome in Patients with Polycystic Ovary Syndrome in Iran. Int. J. Fertil. Steril..

[35] Khorshidi A., Azami M., Tardeh S., Tardeh Z. (2019). The Prevalence of Metabolic Syndrome in Patients with Polycystic Ovary Syndrome: A Systematic Review and Meta-Analysis. Diabetes Metab. Syndr. Clin. Res. Rev..

[36] Shruthi S., Lakshmi G., S M.T., Washington J.K. (2025). Prevalence and Predictors of Metabolic Syndrome in Women with Polycystic Ovarian Syndrome: A Cross-Sectional Study. Cureus.

[37] Anjum S., Askari S., Riaz M., Basit A. (2020). Clinical Presentation and Frequency of Metabolic Syndrome in Women with Polycystic Ovary Syndrome: An Experience from a Tertiary Care Hospital in Pakistan. Cureus.

[38] Glass L.M., Hunt C.M., Fuchs M., Su G.L. (2019). Comorbidities and Nonalcoholic Fatty Liver Disease: The Chicken, the Egg, or Both?. Fed. Pract..

[39] Godoy-Matos A.F., Silva Júnior W.S., Valerio C.M. (2020). NAFLD as a Continuum: From Obesity to Metabolic Syndrome and Diabetes. Diabetol. Metab. Syndr..

[40] Jinjuvadia R., Antaki F., Lohia P., Liangpunsakul S. (2017). The Association between Nonalcoholic Fatty Liver Disease and Metabolic Abnormalities in United States Population. J. Clin. Gastroenterol..

[41] Wang D., He B. (2022). Current Perspectives on Nonalcoholic Fatty Liver Disease in Women with Polycystic Ovary Syndrome. Diabetes Metab. Syndr. Obes..

[42] Min H.K., Kapoor A., Fuchs M., Mirshahi F., Zhou H., Maher J., Kellum J., Warnick R., Contos M.J., Sanyal A.J. (2012). Increased Hepatic Synthesis and Dysregulation of Cholesterol Metabolism Is Associated with the Severity of Nonalcoholic Fatty Liver Disease. Cell Metab..

[43] Liu D., Gao X., Pan X.F., Zhou T., Zhu C., Li F., Fan J.G., Targher G., Zhao J. (2023). The Hepato-Ovarian Axis: Genetic Evidence for a Causal Association between Non-Alcoholic Fatty Liver Disease and Polycystic Ovary Syndrome. BMC Med..

[44] Chen Y., Ma L., Ge Z., Pan Y., Xie L. (2022). Key Genes Associated with Non-Alcoholic Fatty Liver Disease and Polycystic Ovary Syndrome. Front. Mol. Biosci..

[45] Asfari M.M., Sarmini M.T., Baidoun F., Al-Khadra Y., Ezzaizi Y., Dasarathy S., McCullough A. (2020). Association of Non-Alcoholic Fatty Liver Disease and Polycystic Ovarian Syndrome. BMJ Open Gastroenterol..

[46] Rađenović S.S., Pupovac M., Andjić M., Bila J., Srećković S., Gudović A., Dragaš B., Radunović N. (2022). Prevalence, Risk Factors, and Pathophysiology of Nonalcoholic Fatty Liver Disease (NAFLD) in Women with Polycystic Ovary Syndrome (PCOS). Biomedicines.

[47] Shahbaz M., Almatooq H., Foucambert P., Esbrand F.D., Zafar S., Panthangi V., Cyril Kurupp A.R., Raju A., Luthra G., Khan S. (2022). A Systematic Review of the Risk of Non-Alcoholic Fatty Liver Disease in Women with Polycystic Ovary Syndrome. Cureus.

[48] Ayonrinde O.T., Mori T.A., Adams L.A., Beilin L.J., Olynyk J.K., Hart R. (2026). MASLD Coexisting with PCOS Increases Cardiometabolic Risk. J. Clin. Endocrinol. Metab..

[49] Maldonado S.S., Grab J., Wang C.W., Huddleston H., Cedars M., Sarkar M. (2022). Polycystic Ovary Syndrome Is Associated with Nonalcoholic Steatohepatitis in Women of Reproductive Age. Hepatol. Commun..

[50] Pandyarajan V., Gish R.G., Alkhouri N., Noureddin M. (2019). Screening for Nonalcoholic Fatty Liver Disease in the Primary Care Clinic. Gastroenterol. Hepatol..

[51] Cusi K., Abdelmalek M.F., Apovian C.M., Balapattabi K., Bannuru R.R., Barb D., Bardsley J.K., Beverly E.A., Corbin K.D., Elsayed N.A. (2025). Metabolic Dysfunction–Associated Steatotic Liver Disease (MASLD) in People with Diabetes: The Need for Screening and Early Intervention. A Consensus Report of the American Diabetes Association. Diabetes Care.

[52] Ullah R., Rauf N., Nabi G., Ullah H., Shen Y., Zhou Y.D., Fu J. (2019). Role of Nutrition in the Pathogenesis and Prevention of Non-Alcoholic Fatty Liver Disease: Recent Updates. Int. J. Biol. Sci..

[53] Ul-Haq I., Nadeem M., Sharma A., Mugabi R., Waseem M., Nayik G.A. (2025). Beyond Medication: Unveiling the Role of Diet and Lifestyle in Fatty Liver Disease Management. Hum. Nutr. Metab..

[54] Gautam R., Maan P., Jyoti A., Kumar A., Malhotra N., Arora T. (2025). The Role of Lifestyle Interventions in PCOS Management: A Systematic Review. Nutrients.

[55] Shishehgar F., Mirmiran P., Rahmati M., Tohidi M., Ramezani Tehrani F. (2019). Does a Restricted Energy Low Glycemic Index Diet Have a Different Effect on Overweight Women with or without Polycystic Ovary Syndrome?. BMC Endocr. Disord..

[56] Marsh K.A., Steinbeck K.S., Atkinson F.S., Petocz P., Brand-Miller J.C. (2010). Effect of a Low Glycemic Index Compared with a Conventional Healthy Diet on Polycystic Ovary Syndrome. Am. J. Clin. Nutr..

[57] Rooholahzadegan F., Arefhosseini S., Tutunchi H., Badali T., Khoshbaten M., Ebrahimi-Mameghani M. (2023). The Effect of DASH Diet on Glycemic Response, Meta-Inflammation and Serum LPS in Obese Patients with NAFLD: A Double-Blind Controlled Randomized Clinical Trial. Nutr. Metab..

[58] Asemi Z., Samimi M., Tabassi Z., Shakeri H., Sabihi S.S., Esmaillzadeh A. (2014). Effects of DASH Diet on Lipid Profiles and Biomarkers of Oxidative Stress in Overweight and Obese Women with Polycystic Ovary Syndrome: A Randomized Clinical Trial. Nutrition.

[59] Azadi-Yazdi M., Karimi-Zarchi M., Salehi-Abargouei A., Fallahzadeh H., Nadjarzadeh A. (2017). Effects of Dietary Approach to Stop Hypertension Diet on Androgens, Antioxidant Status and Body Composition in Overweight and Obese Women with Polycystic Ovary Syndrome: A Randomised Controlled Trial. J. Hum. Nutr. Diet..

[60] Khalatbari-Soltani S., Marques-Vidal P., Imamura F., Forouhi N.G. (2020). Prospective Association between Adherence to the Mediterranean Diet and Hepatic Steatosis: The Swiss CoLaus Cohort Study. BMJ Open.

[61] Barrea L., Arnone A., Annunziata G., Muscogiuri G., Laudisio D., Salzano C., Pugliese G., Colao A., Savastano S. (2019). Adherence to the Mediterranean Diet, Dietary Patterns and Body Composition in Women with Polycystic Ovary Syndrome (PCOS). Nutrients.

[62] Ryan M.C., Itsiopoulos C., Thodis T., Ward G., Trost N., Hofferberth S., O’Dea K., Desmond P.V., Johnson N.A., Wilson A.M. (2013). The Mediterranean Diet Improves Hepatic Steatosis and Insulin Sensitivity in Individuals with Non-Alcoholic Fatty Liver Disease. J. Hepatol..

[63] Manta A., Paschou S.A., Isari G., Mavroeidi I., Kalantaridou S., Peppa M. (2023). Glycemic Index and Glycemic Load Estimates in the Dietary Approach of Polycystic Ovary Syndrome. Nutrients.

[64] Low-Glycemic Index Diet: What’s Behind the Claims?—Mayo Clinic. https://www.mayoclinic.org/healthy-lifestyle/nutrition-and-healthy-eating/in-depth/low-glycemic-index-diet/art-20048478.

[65] Saadati N., Haidari F., Barati M., Nikbakht R., Mirmomeni G., Rahim F. (2021). The Effect of Low Glycemic Index Diet on the Reproductive and Clinical Profile in Women with Polycystic Ovarian Syndrome: A Systematic Review and Meta-Analysis. Heliyon.

[66] Kazemi M., Hadi A., Pierson R.A., Lujan M.E., Zello G.A., Chilibeck P.D. (2020). Effects of Dietary Glycemic Index and Glycemic Load on Cardiometabolic and Reproductive Profiles in Women with Polycystic Ovary Syndrome: A Systematic Review and Meta-Analysis of Randomized Controlled Trials. Adv. Nutr..

[67] DASH—DASH Eating Plan|NHLBI, NIH. https://www.nhlbi.nih.gov/health/dash-eating-plan.

[68] Chiavaroli L., Viguiliouk E., Nishi S.K., Mejia S.B., Rahelić D., Kahleová H., Salas-Salvadó J., Kendall C.W.C., Sievenpiper J.L. (2019). DASH Dietary Pattern and Cardiometabolic Outcomes: An Umbrella Review of Systematic Reviews and Meta-Analyses. Nutrients.

[69] Valipour G., Esmaillzadeh A., Azadbakht L., Afshar H., Hassanzadeh A., Adibi P. (2015). Adherence to the DASH Diet in Relation to Psychological Profile of Iranian Adults. Eur. J. Nutr..

[70] What Is the Mediterranean Diet?|American Heart Association. https://www.heart.org/en/healthy-living/healthy-eating/eat-smart/nutrition-basics/mediterranean-diet.

[71] Widmer R.J., Flammer A.J., Lerman L.O., Lerman A. (2015). The Mediterranean Diet, Its Components, and Cardiovascular Disease. Am. J. Med..

[72] Abiemo E.E., Alonso A., Nettleton J.A., Steffen L.M., Bertoni A.G., Jain A., Lutsey P.L. (2013). Relationships of the Mediterranean Dietary Pattern with Insulin Resistance and Diabetes Incidence in the Multi-Ethnic Study of Atherosclerosis (MESA). Br. J. Nutr..

[73] Boghossian N.S., Yeung E.H., Mumford S.L., Zhang C., Gaskins A.J., Wactawski-Wende J., Schisterman E.F. (2013). Adherence to the Mediterranean Diet and Body Fat Distribution in Reproductive Aged Women. Eur. J. Clin. Nutr..

[74] Estruch R., Ros E., Salas-Salvadó J., Covas M.-I., Corella D., Arós F., Gómez-Gracia E., Ruiz-Gutiérrez V., Fiol M., Lapetra J. (2018). Primary Prevention of Cardiovascular Disease with a Mediterranean Diet Supplemented with Extra-Virgin Olive Oil or Nuts. N. Engl. J. Med..

[75] Koloverou E., Esposito K., Giugliano D., Panagiotakos D. (2014). The Effect of Mediterranean Diet on the Development of Type 2 Diabetes Mellitus: A Meta-Analysis of 10 Prospective Studies and 136,846 Participants. Metabolism.

[76] Magagnini M.C., Condorelli R.A., Cimino L., Cannarella R., Aversa A., Calogero A.E., La Vignera S. (2022). Does the Ketogenic Diet Improve the Quality of Ovarian Function in Obese Women?. Nutrients.

[77] Goss A.M., Chandler-Laney P.C., Ovalle F., Goree L.L., Azziz R., Desmond R.A., Wright Bates G., Gower B.A. (2014). Effects of a Eucaloric Reduced-Carbohydrate Diet on Body Composition and Fat Distribution in Women with PCOS. Metabolism.

[78] Mavropoulos J.C., Yancy W.S., Hepburn J., Westman E.C. (2005). The Effects of a Low-Carbohydrate, Ketogenic Diet on the Polycystic Ovary Syndrome: A Pilot Study. Nutr. Metab..

[79] Khazaei Y., Dehghanseresht N., Mousavi S.E., Nazari M., Salamat S., Asbaghi O., Mansoori A. (2023). Association Between Protein Intake from Different Animal and Plant Origins and the Risk of Non-Alcoholic Fatty Liver Disease: A Case-Control Study. Clin. Nutr. Res..

[80] Kasim-Karakas S.E., Almario R.U., Cunningham W. (2009). Effects of Protein versus Simple Sugar Intake on Weight Loss in Polycystic Ovary Syndrome (According to the National Institutes of Health Criteria). Fertil. Steril..

[81] Zumbro E.L., Rao M., Balcom-Luker S., Broughton K.S., LeMieux M.J. (2021). Whey Protein Supplementation Improves the Glycemic Response and May Reduce Non-Alcoholic Fatty Liver Disease Related Biomarkers in Women with Polycystic Ovary Syndrome (PCOS). Nutrients.

[82] Khani B., Mehrabian F., Khalesi E., Eshraghid A. (2011). Effect of Soy Phytoestrogen on Metabolic and Hormonal Disturbance of Women with Polycystic Ovary Syndrome. J. Res. Med. Sci..

[83] Yahay M., Heidari Z., Allameh Z., Amani R. (2021). The Effects of Canola and Olive Oils Consumption Compared to Sunflower Oil, on Lipid Profile and Hepatic Steatosis in Women with Polycystic Ovarian Syndrome: A Randomized Controlled Trial. Lipids Health Dis..

[84] Nigam P., Bhatt S., Misra A., Chadha D.S., Vaidya M., Dasgupta J., Pasha Q.M.A. (2014). Effect of a 6-Month Intervention with Cooking Oils Containing a High Concentration of Monounsaturated Fatty Acids (Olive and Canola Oils) Compared with Control Oil in Male Asian Indians with Nonalcoholic Fatty Liver Disease. Diabetes Technol. Ther..

[85] Scorletti E., Bhatia L., McCormick K.G., Clough G.F., Nash K., Hodson L., Moyses H.E., Calder P.C., Byrne C.D. (2014). Effects of Purified Eicosapentaenoic and Docosahexaenoic Acids in Nonalcoholic Fatty Liver Disease: Results from the WELCOME* Study. Hepatology.

[86] Cussons A.J., Watts G.F., Mori T.A., Stuckey B.G.A. (2009). Omega-3 Fatty Acid Supplementation Decreases Liver Fat Content in Polycystic Ovary Syndrome: A Randomized Controlled Trial Employing Proton Magnetic Resonance Spectroscopy. J. Clin. Endocrinol. Metab..

[87] Khani B., Mardanian F., Fesharaki S.J. (2017). Omega-3 Supplementation Effects on Polycystic Ovary Syndrome Symptoms and Metabolic Syndrome. J. Res. Med. Sci..

[88] Soltani S., Jayedi A., Abdollahi S., Vasmehjani A.A., Meshkini F., Shab-Bidar S. (2023). Effect of Carbohydrate Restriction on Body Weight in Overweight and Obese Adults: A Systematic Review and Dose–Response Meta-Analysis of 110 Randomized Controlled Trials. Front. Nutr..

[89] Shariq A., Khan S., Usmani S.U.R. (2025). The Role of Dietary Protein in Mitigating the Risk of Nonalcoholic Fatty Liver Disease. Nutr. Rev..

[90] Patil P., Mandal S., Tomar S.K., Anand S. (2015). Food Protein-Derived Bioactive Peptides in Management of Type 2 Diabetes. Eur. J. Nutr..

[91] Brandelli A., Daroit D.J., Corrêa A.P.F. (2015). Whey as a Source of Peptides with Remarkable Biological Activities. Food Res. Int..

[92] Siriwardhana N., Kalupahana N.S., Cekanova M., LeMieux M., Greer B., Moustaid-Moussa N. (2013). Modulation of Adipose Tissue Inflammation by Bioactive Food Compounds. J. Nutr. Biochem..

[93] Siriwardhana N., Kalupahana N.S., Fletcher S., Xin W., Claycombe K.J., Quignard-Boulange A., Zhao L., Saxton A.M., Moustaid-Moussa N. (2012). N-3 and n-6 Polyunsaturated Fatty Acids Differentially Regulate Adipose Angiotensinogen and Other Inflammatory Adipokines in Part via NF-ΚB-Dependent Mechanisms. J. Nutr. Biochem..

[94] Siriwardhana N., Kalupahana N.S., Moustaid-Moussa N. (2012). Health Benefits of N-3 Polyunsaturated Fatty Acids: Eicosapentaenoic Acid and Docosahexaenoic Acid. Adv. Food Nutr. Res..

[95] Flachs P., Rossmeisl M., Bryhn M., Kopecky J. (2009). Cellular and Molecular Effects of N-3 Polyunsaturated Fatty Acids on Adipose Tissue Biology and Metabolism. Clin. Sci..

[96] Kalupahana N.S., Moustaid-Moussa N., Claycombe K.J. (2012). Immunity as a Link between Obesity and Insulin Resistance. Mol. Asp. Med..

[97] Musa-Veloso K., Binns M.A., Kocenas A.C., Poon T., Elliot J.A., Rice H., Oppedal-Olsen H., Lloyd H., Lemke S. (2010). Long-Chain Omega-3 Fatty Acids Eicosapentaenoic Acid and Docosahexaenoic Acid Dose-Dependently Reduce Fasting Serum Triglycerides. Nutr. Rev..

[98] Stirban A., Nandrean S., Götting C., Tamler R., Pop A., Negrean M., Gawlowski T., Stratmann B., Tschoepe D. (2010). Effects of n–3 Fatty Acids on Macro- and Microvascular Function in Subjects with Type 2 Diabetes Mellitus. Am. J. Clin. Nutr..

[99] Kelley D.S., Adkins Y., Woodhouse L.R., Swislocki A., MacKey B.E., Siegel D. (2012). Docosahexaenoic Acid Supplementation Improved Lipocentric but Not Glucocentric Markers of Insulin Sensitivity in Hypertriglyceridemic Men. Metab. Syndr. Relat. Disord..

[100] Sarbolouki S., Javanbakht M.H., Derakhshanian H., Hosseinzadeh P., Zareei M., Hashemi S.B., Dorosty A.R., Eshraghian M.R., Djalali M. (2013). Eicosapentaenoic Acid Improves Insulin Sensitivity and Blood Sugar in Overweight Type 2 Diabetes Mellitus Patients: A Double-Blind Randomised Clinical Trial. Singap. Med. J..

[101] Rivellese A.A., Maffettone A., Iovine C., Di Marino L., Annuzzi G., Mancini M., Riccardi G. (1996). Long-Term Effects of Fish Oil on Insulin Resistance and Plasma Lipoproteins in NIDDM Patients with Hypertriglyceridemia. Diabetes Care.

[102] Kabir M., Skurnik G., Naour N., Pechtner V., Meugnier E., Rome S., Quignard-Boulangé A., Vidal H., Slama G., Clément K. (2007). Treatment for 2 Mo with N-3 Polyunsaturated Fatty Acids Reduces Adiposity and Some Atherogenic Factors but Does Not Improve Insulin Sensitivity in Women with Type 2 Diabetes: A Randomized Controlled Study. Am. J. Clin. Nutr..

[103] Hendrich S. (2010). (N-3) Fatty Acids: Clinical Trials in People with Type 2 Diabetes. Adv. Nutr..

[104] Xia Y., Wang Y., Cui M., Su D. (2021). Efficacy of Omega-3 Fatty Acid Supplementation on Cardiovascular Risk Factors in Patients with Polycystic Ovary Syndrome: A Systematic Review and Meta-Analysis. Ann. Palliat. Med..

[105] Javed Z., Papageorgiou M., Deshmukh H., Kilpatrick E.S., Mann V., Corless L., Abouda G., Rigby A.S., Atkin S.L., Sathyapalan T. (2019). A Randomized, Controlled Trial of Vitamin D Supplementation on Cardiovascular Risk Factors, Hormones, and Liver Markers in Women with Polycystic Ovary Syndrome. Nutrients.

[106] Sharifi N., Amani R., Hajiani E., Cheraghian B. (2014). Does Vitamin D Improve Liver Enzymes, Oxidative Stress, and Inflammatory Biomarkers in Adults with Non-Alcoholic Fatty Liver Disease? A Randomized Clinical Trial. Endocrine.

[107] Nordio M., Basciani S., Camajani E. (2019). The 40:1 Myo-Inositol/D-Chiro-Inositol Plasma Ratio Is Able to Restore Ovulation in PCOS Patients: Comparison with Other Ratios. Eur. Rev. Med. Pharmacol. Sci..

[108] Mellonie P., Manivannan A., Thangaraj P., Logeswari B.M. (2024). The Effectiveness of Myo-Inositol in Women with Polycystic Ovary Syndrome: A Prospective Clinical Study. Cureus.

[109] Arefhosseini S., Roshanravan N., Asghari S., Tutunchi H., Ebrahimi-Mameghani M. (2023). Expression of Inflammatory Genes, WBC-Derived Inflammatory Biomarkers and Liver Function Indices: Effects of Myo-Inositol Supplementation in Obese Patients with NAFLD. J. Funct. Foods.

[110] Arefhosseini S., Roshanravan N., Tutunchi H., Rostami S., Khoshbaten M., Ebrahimi-Mameghani M. (2023). Myo-Inositol Supplementation Improves Cardiometabolic Factors, Anthropometric Measures, and Liver Function in Obese Patients with Non-Alcoholic Fatty Liver Disease. Front. Nutr..

[111] Izadi A., Ebrahimi S., Shirazi S., Taghizadeh S., Parizad M., Farzadi L., Gargari B.P. (2019). Hormonal and Metabolic Effects of Coenzyme Q10 and/or Vitamin E in Patients with Polycystic Ovary Syndrome. J. Clin. Endocrinol. Metab..

[112] Izadi A., Shirazi S., Taghizadeh S., Gargari B.P. (2019). Independent and Additive Effects of Coenzyme Q10 and Vitamin E on Cardiometabolic Outcomes and Visceral Adiposity in Women with Polycystic Ovary Syndrome. Arch. Med. Res..

[113] Sanyal A.J., Chalasani N., Kowdley K.V., McCullough A., Diehl A.M., Bass N.M., Neuschwander-Tetri B.A., Lavine J.E., Tonascia J., Unalp A. (2010). Pioglitazone, Vitamin E, or Placebo for Nonalcoholic Steatohepatitis. N. Engl. J. Med..

[114] Stipanuk M., Caudill M. (2012). Biochemical, Physiological, and Molecular Aspects of Human Nutrition.

[115] Comerford K.B., Pasin G. (2016). Emerging Evidence for the Importance of Dietary Protein Source on Glucoregulatory Markers and Type 2 Diabetes: Different Effects of Dairy, Meat, Fish, Egg, and Plant Protein Foods. Nutrients.

[116] Chhetri D.R. (2019). Myo-Inositol and Its Derivatives: Their Emerging Role in the Treatment of Human Diseases. Front. Pharmacol..

[117] Roseff S., Montenegro M. (2020). Inositol Treatment for PCOS Should Be Science-Based and Not Arbitrary. Int. J. Endocrinol..

[118] Vitamin E—Mayo Clinic. https://www.mayoclinic.org/drugs-supplements-vitamin-e/art-20364144.

[119] Vitamin E—Health Professional Fact Sheet. https://ods.od.nih.gov/factsheets/VitaminE-HealthProfessional/.

[120] Kowalska A. (2024). The Vitamin E: Overview of History of Discovery, Mechanism of Action, Role and Deficiency. Prospect. Pharm. Sci..

[121] Banini B.A., Sanyal A.J. (2019). Vitamin E in Nonalcoholic Fatty Liver Disease. Vitamin E in Human Health.

[122] Steatotic Liver Disease: Cutting Through the Fat|AASLD. https://www.aasld.org/liver-fellow-network/core-series/back-basics/steatotic-liver-disease-cutting-through-fat.

[123] Tefagh G., Payab M., Qorbani M., Sharifi F., Sharifi Y., Ebrahimnegad Shirvani M.S., Pourghazi F., Atlasi R., Shadman Z., Rezaei N. (2022). Effect of Vitamin E Supplementation on Cardiometabolic Risk Factors, Inflammatory and Oxidative Markers and Hormonal Functions in PCOS (Polycystic Ovary Syndrome): A Systematic Review and Meta-analysis. Sci. Rep..

[124] Rao M., Broughton K.S., LeMieux M.J. (2020). Cross-Sectional Study on the Knowledge and Prevalence of PCOS at a Multiethnic University. Prog. Prev. Med..

[125] Gibson-Helm M., Teede H., Dunaif A., Dokras A. (2017). Delayed Diagnosis and a Lack of Information Associated with Dissatisfaction in Women with Polycystic Ovary Syndrome. J. Clin. Endocrinol. Metab..

[126] Teede H.J., Khomami M.B., Morman R., Laven J.S.E., Joham A.E., Costello M.F., Patil M., Rees D.A., Berry L., Cree M.G. (2026). Polyendocrine Metabolic Ovarian Syndrome, the New Name for Polycystic Ovary Syndrome: A Multistep Global Consensus Process. Lancet.

[127] Shang Y., Nasr P., Widman L., Hagström H. (2022). Risk of Cardiovascular Disease and Loss in Life Expectancy in NAFLD. Hepatology.

[128] MASLD (Nonalcoholic Fatty Liver Disease). https://my.clevelandclinic.org/health/diseases/22437-non-alcoholic-fatty-liver-disease.

[129] Fibrosis: Development. https://liverfoundation.org/about-your-liver/how-liver-diseases-progress/fibrosis-scarring/.

[130] Engmann L., Jin S., Sun F., Legro R.S., Polotsky A.J., Hansen K.R., Coutifaris C., Diamond M.P., Eisenberg E., Zhang H. (2017). Racial and Ethnic Differences in the Polycystic Ovary Syndrome (PCOS) Metabolic Phenotype. Am. J. Obstet. Gynecol..

[131] Rich N.E., Oji S., Mufti A.R., Browning J.D., Parikh N.D., Odewole M., Mayo H., Singal A.G. (2017). Racial and Ethnic Disparities in Non-Alcoholic Fatty Liver Disease Prevalence, Severity, and Outcomes in the United States: A Systematic Review and Meta-Analysis. Clin. Gastroenterol. Hepatol..

[132] Manoogian E.N.C., Laferrère B. (2023). Time-Restricted Eating: What We Know and Where the Field Is Going. Obesity.

[133] Vidya Bharathi R., Swetha S., Neerajaa J., Varsha Madhavica J., Janani D.M., Rekha S.N., Ramya S., Usha B. (2025). The Impact of Intermittent Fasting on Fertility: A Focus on Polycystic Ovary Syndrome and Reproductive Outcomes in Women—A Systematic Review. Metabol. Open.

[134] Berciano S., Figueiredo J., Brisbois T.D., Alford S., Koecher K., Eckhouse S., Ciati R., Kussmann M., Ordovas J.M., Stebbins K. (2022). Precision Nutrition: Maintaining Scientific Integrity While Realizing Market Potential. Front. Nutr..

[135] Mogna-Peláez P., Riezu-Boj J.I., Milagro F.I., Herrero J.I., Elorz M., Benito-Boillos A., Tobaruela-Resola A.L., Tur J.A., Martínez J.A., Abete I. (2024). Inflammatory Markers as Diagnostic and Precision Nutrition Tools for Metabolic Dysfunction-Associated Steatotic Liver Disease: Results from the Fatty Liver in Obesity Trial. Clin. Nutr..

[136] Shamasbi S.G., Ghanbari-Homayi S., Mirghafourvand M. (2019). The Effect of Probiotics, Prebiotics, and Synbiotics on Hormonal and Inflammatory Indices in Women with Polycystic Ovary Syndrome: A Systematic Review and Meta-Analysis. Eur. J. Nutr..

[137] Rocha A.L.L., Faria L.C., Guimarães T.C.M., Moreira G.V., Cândido A.L., Couto C.A., Reis F.M. (2017). Non-Alcoholic Fatty Liver Disease in Women with Polycystic Ovary Syndrome: Systematic Review and Meta-Analysis. J. Endocrinol. Investig..

